# Influence of the aromatic surface on the capacity of adsorption of VOCs by magnetite supported organic–inorganic hybrids†

**DOI:** 10.1039/c9ra04490f

**Published:** 2019-08-05

**Authors:** María de las Nieves Piña, María Susana Gutiérrez, Mario Panagos, Paulino Duel, Alberto León, Jeroni Morey, David Quiñonero, Antonio Frontera

**Affiliations:** Department of Chemistry, Universitat de les Illes Balears Crta. de Valldemossa km 7.5 07122 Palma de Mallorca Spain toni.frontera@uib.es

## Abstract

It has been recently evidenced that hybrid magnetic nanomaterials based on perylene diimide (PDI) dopamine and iron oxide nanoparticles are useful for the adsorption and determination of volatile organic compounds (VOCs). However, NDI compounds are expensive and difficult to handle compared to smaller size diimides. Therefore, in this manuscript a combined experimental and theoretical investigation is reported including the analysis of the effect of changing the aromatic surface on the ability of these magnetite supported organic–inorganic hybrid nanoparticles (NPs) to adsorb several aromatic and non-aromatic VOCs. In particular, two new hybrid Fe_3_O_4_NPs are synthesized and characterized where the size of organic PDI dopamine linker is progressively reduced to naphthalene diimide (NDI) and pyromellitic diimide (PMDI). These materials were utilized to fill two sorbent tubes in series. Thermal desorption (TD) combined with capillary gas chromatography (GC)/flame detector (FID) was used to analyze both front and back tubes. Adsorption values (defined as % VOCs found in the front tube) were determined for a series of VOCs. The binding energies (DFT-D3 calculations) of VOC–Fe_3_O_4_NP complexes were also computed to correlate the electron-accepting ability of the arylene diimide (PDI, NDI or PMDI) with the adsorption capacity of the different tubes. The prepared hybrids can be easily separated magnetically and showed great reusability.

## Abstract 2

The effect of changing the size of the aromatic surface (perylene diimide, naphthalene diimide and pyromellitic diimide) upon the ability of hybrid magnetic nanoparticles to adsorb VOCs is rationalized in this work.

## Graphical abstract

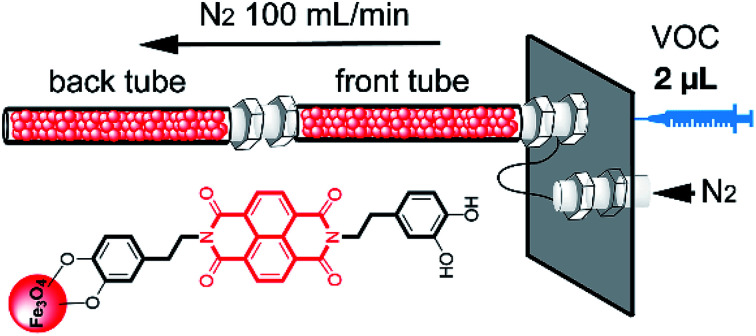

## Introduction

Volatile organic compounds (VOCs) are inherent to modern human life. For instance, they are used as markers in medicine^1^ and industry.^2^ Moreover, they are used as indicators for the presence of other substances. The investigation of VOCs is intensive in many areas like environmental research,^3^ medicine,^4^ and even in military applications.^5^ The analysis and quantification of VOCs is needed to control the quality of biomaterials^6^ and they are also used as markers of microbial contamination in the case of bacteria and fungi.^7^ Moreover, VOCs are also normally used as markers in food industry for controlling the quality of raw materials.^8^

Regrettably, the number of endogenic pollutants and their sources continuously rises. Therefore, it is very important to actively monitor pollutants that are generated not only from agricultural but also from personal care products and pharmaceuticals, such as birth control pills, painkillers and antibiotics.^9–11^ Consequently it is even more important to investigate new methodologies and develop low cost techniques for the monitoring of air,^12^ since VOCs are ubiquitously present in our indoor daily lives. In fact, humans are constantly exposed to smells from many sources (fragrances, furnishings, cleaners, disinfectants, printers, glues, *etc.*). VOCs are then considered essential to assess the quality of the air in indoor environments.^13–15^

For several decades, studies on application of gas sensors for detection of VOCs have been carried out.^16^ There are several types of chemically sensitive sensors for VOCs, including metal oxides, conducting polymers, sensors based on acoustic wave propagation, *etc.*^17^ Nowadays electronic noses with a large number of resistive sensors based on statistical methods are used to facilitate the recognition of VOCs. The commercially available electronic noses are built with conducting polymer and metal oxide sensors.^18^ A serious drawback of this type of sensors^19^ is the signal degradation over time and the complex composition of the sampling matrices.^20^ Some enhancement is achieved by utilizing multiple sensors^20*c*^ or sensor arrays integrated on a multimodal sensing platform^21^ that facilitates a precise detection in real-time.^22,23^ The progress in micromachining and nanotechnology allows building sensors of small size.^24^ As a matter of fact, the utilization of nanomaterial improves the sensing performance and facilitates the generation of new multisensing arrays.^25,26^ There is a growing interest in the utilization of different types of nanomaterial for the design of new diagnostic tools. The main advantages of these materials are the high surface/volume ratio, fast response time and easy recovery and reutilization.^27^ For instance, Li *et al.*^28^ have reported the utilization of polythiophene nanostructured for the detection of VOCs that exhibited enhanced discrimination compared to standard diagnostic tools.

Compared to other materials, the main advantages of iron oxide (Fe_3_O_4_) magnetic NPs follow: (i) economic and easy to handle and prepare; (ii) versatile *via* functionalization and (iii) very easy to recuperate by means of an external magnetic field.^29^ As a matter of fact, it has been recently demonstrated that hybrid nanomaterials^30^ consisting of chitosan-coated magnetite nanocomposite are very convenient catalysts because of their reusability. The main advantage of these catalysts is the presence of magnetite core since it allows an easy recovery and reuse in catalytic reactions. Therefore, their use is convenient in the sense of reuse and sustainability with the environment.^31^ In fact, our group has recently used a PDI-dopamine derivative to functionalize hybrid nanoparticles (Fe_3_O_4_NP), which were utilized to fill sorbent tubes. These tubes were used and reused more than 200 times for the adsorption and quantification of aromatic VOCs.^32^

**Scheme 1:**
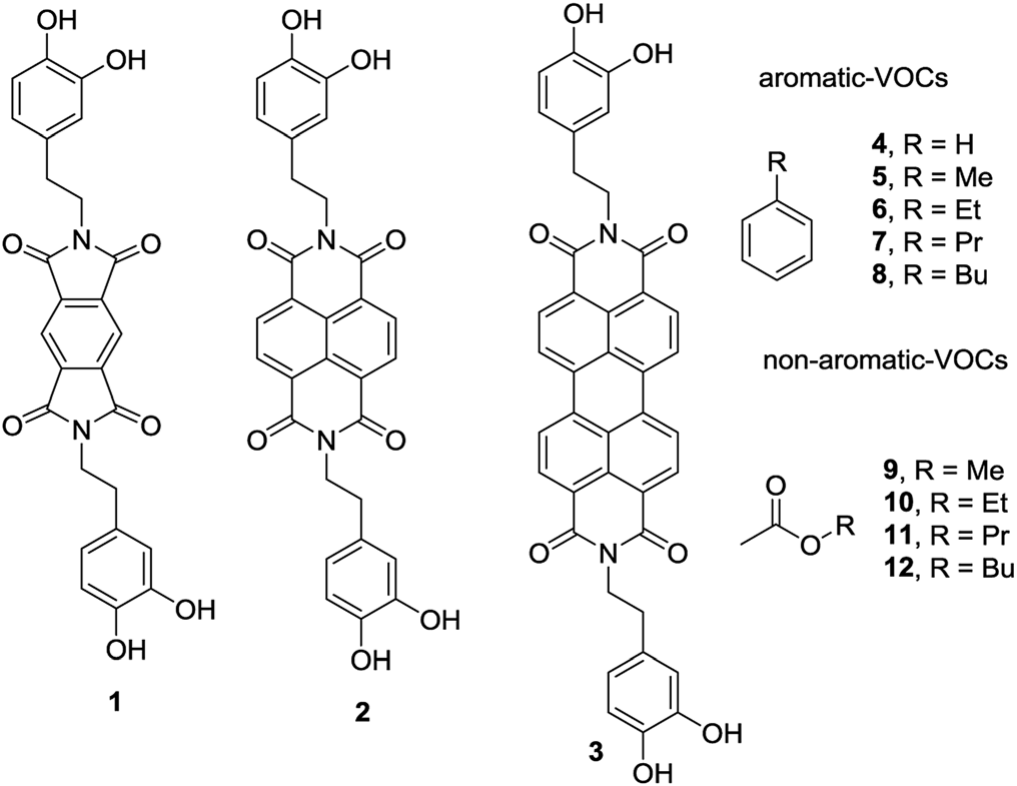
PMDI (1), NDI (2) and PDI (3) derivatives used to functionalize the Fe_3_O_4_NP. VOCs 4 to 9 used in this study.

In this new investigation, the possibility of using smaller and cheaper substitutes of PDI (see Scheme 1) is explored, which are pyromellitic diimide (PMDI) 1 and naphthalene diimide (NDI) 2 to adsorb and quantify a series of VOCs. The results are compared with those obtained using the PDI-dopamine derivative. It is worth mentioning that dopamine containing pyromellitic diimide has been successfully used for the molecular recognition of electron rich guests.^33^ The interaction energies of the VOCs with compounds 1–3 were initially computed by using theoretical calculations (DFT-D3). Moreover, sorbent tubes filled with the three hybrid nanoparticles were built. Then a simple analytical method based on thermal desorption (TD) coupled to gas chromatography (GC) and FID detection was used and validated for the determination of the VOCs tested herein (compounds 4–12, see Scheme 1). The two families of VOCs selected (aromatic and non-aromatic) present an increasing size aliphatic chain as substituent. The sorbent tubes were filled with PDI, NDI and PMDI-based magnetite supported organic–inorganic hybrids and their ability to adsorb the VOCs was determined and compared. The different affinities showed by the hybrid nanoparticles for the adsorption and quantification of the VOCs was also compared with the DFT calculations that are in agreement with the experimental results. The increasing number of C–H⋯π interactions on going from compound 4 to 8 are responsible for the higher adsorption of the VOCs and the alkyl chain grows.

## Methods

The synthesis and characterization of the PMDI-Fe_3_O_4_ nanoparticles used herein have been carried out using the methodology described in the literature by others.^34^ The synthesis and characterization of the PDI-Fe_3_O_4_ hybrid nanoparticles have been previously described by us.^32^ Reagents and solvents required for the synthesis of the new NDI–Fe_3_O_4_ nanoparticles were purchased from Sigma-Aldrich and used without further purification. The magnetic nanoparticle functionalization reactions were carried out in a Biotage Initiator Classic Microwave Synthesizer microwave reactor with a power of 400 W. The magnets used in magnetic decantation are NdFeB magnets, with an N52 magnetic degree. The functionalized nanoparticles are thermally stable up to 450 °C, therefore they are suitable for utilization in the experiments detailed below, since the maximum temperature used in the adsorption–desorption apparatus is 375 °C. Remarkably, no degradation of the magnetic nanoparticles even after 200 repetition of the experiment is observed. The VOC analytes were purchased from Sigma-Aldrich and Fluka (Buchs, Switzerland) and their purity was higher than 98% in all cases. The solvent was purchased from Scharlau (chromatographic quality). The glass tubes (6 mm of diameter and 90 mm length) and the unsilanized glass wool were purchased from Supelco (Bellefonte, PA, USA).

### Synthesis of naphthalendiimide-dopamine (NDI-DA)

1 mmol of 1,4,5,8-naphthalentetracarboxylic dianhydride is dissolved in 25 mL of DMF, in a round-bottomed flask, with a 2 mL of Et_3_N. After total solution, 2 mmol of dopamine hydrochloride dissolved in DMF were added. The reaction mixture was brought to constant reflux for 2 hours. After the reaction, most of the DMF was removed in a rotary evaporator, and then 100 mL of cold H_2_O were added to precipitate the product. Before that, the solid was filtered under vacuum, washed with cold water (3 × 10 mL) and air-dried. As a result, the final product was obtained with a good yield (80%). Molecular weight: 538.51 g mol^−1^. Melting point: >200 °C. ^1^H-NMR: (DMSO-d_6_) *δ*: 8.83 (s, 4H), 8.67 (s, 4H), 6.65 (m, 6H), 4.18 (t, 4H) and 2.75 (t, 4H) ppm. ^13^C (DMSO-d_6_) *δ*: 162.44; 145.22; 143.79; 130.45; 129.30; 126.28; 119.26; 115.96; 115.64; 41.82 and 32.81 ppm. FTIR (KBr): 3386, 2968, 1699, 1654, 1336 and 768 cm^−1^.

### Functionalization of nanoparticles with NDI-DA

One drop of 1 M NaOH and 1 mL of FeNP (10 mg mL^−1^) were added to a solution of 0.025 mmol of NDI-DA in 4 mL of H_2_O. The mixture was sealed in a 5 mL microwave flask, sonicated with an ultrasound for 5 minutes and then introduced in a microwave reactor at 120 °C and 3 bar for 30 minutes. The resulting mixture containing the functionalized FeNPs was decanted magnetically and washed firstly with 5 mL of ethanol and secondly with 5 mL of H_2_O. The final product was dispersed in 10 mL of H_2_O at a concentration of 1 mg mL^−1^. FTIR (KBr): 3475, 3381, 2924, 2852, 1704, 1656, 1581, 1455, 1337, 1243, 1170, 1119, 1028, 880, 769, 692 and 599 cm^−1^. BET specific surface area: 79.07 m^2^ g^−1^. Potential *Z* (*ζ*) (H_2_O, 25 °C): −38.3 mV. Average diameter (SEM): 60.46 nm. Hydrodynamic diameter (*D*_h_) (H_2_O, 25 °C): 300.6 nm (see ESI† for further details, including SEM micrographic images, DLS size distribution and zeta potential of all hybrid nanoparticles used in this work).

### Standard solutions

For each VOC used in this study freshly prepared stock standard solutions on the day of use were stored at 4 °C in darkness in 1.5 mL flasks. The standard solutions were prepared using these mother solutions by using methanol as diluent. The concentrations of the standard solutions were: 0.140, 0.280, 0.420, 0.560, 0.700, 0.840 and 0.98 μL VOC/μL with a final volume of 1000 μL. The quantity injected was 2 μL of standard solution of 0.7 μL VOC/μL (1.4 μL VOC) for the determination of the percentage of retention.

### Tubes filled with magnetite supported organic–inorganic hybrids

A single tube was used for the generation of the calibration plots and two tubes connected in series were used to calculate the percentage of retention of the analyte in the functionalized nanoparticles. 450 mg of hybrid nanoparticles were introduced in each tube and a periodical test was done to both tubes to check that were able to give the same analytical result when doped with the same quantity of a given VOC. After total desorption, a verification that both tubes are perfectly clean was carried out. Before the first use, a thermal cleaning was performed using the following protocol: 250 °C for 20 min, 300 °C for 20 min, 330 °C for 20 min, 350 °C for 20 min and 375 °C for 20 min under a flow rate of dry N_2_ of 70 mL min^−1^. For successive experiments, pre-conditioning for 20 min at 375 °C was used. After conditioning, they were immediately sealed with Swagelock end caps fitted with PTFE ferrules and stored in closed plastic boxes filled with desiccant material. Samples used for the doping and desorption experiments were freshly prepared immediately after the preparation of the tubes. The commercial doping device supplied by the instrument manufacturer was employed for the doping of the tubes. A manual injection under a flow rate of dry N_2_ of 100 mL min^−1^ of the VOC was carried out. This is done through a Swagelock adapter (stainless steel) that connects the tube without any possibility of leakage. The constant N_2_ flow guarantees the homogeneity of the sample through the sorbent. For the sake of reproducibility, 1 μL of VOC solution was always used and a doping time of 5 min was applied. During the first minute, the syringe was kept in the injector in order to maintain the flux unperturbed.

### Analytical instrumentation and procedure

The VOC analyses were carried out using a manual injection (Unity 2 model, Markes) thermal desorption (TD) coupled to a gas chromatograph with a FID detector (6890A model, Agilent Technologies). The first step is an initial pre-desorption during 0.1 min and a flux of 20 mL min^−1^. Subsequently, a primary desorption is performed at 300 °C during 10 min, with a split of 4 mL min^−1^. During this time period, the analyte that was previously adsorbed in the tube is concentrated in a criofocusing trap that contains 30 mg of Tenax TA, and kept at −30 °C. Afterwards, the trap is quickly heated from −30 °C to 300 °C and kept at the final temperature during 10 min and, thus, the secondary desorption takes place. In this step the analyte is sent to the gas chromatograph using a split with a flux of 7 mL min^−1^ and finally injected to a capillary column (DB-624, 60 m × 0.25 mm × 1.4 μm, Agilent Technologies) through a line at 200 °C. The oven temperature was initially set at 40 °C during 1 min and progressively increased (rate of 6 °C min^−1^) until 230 °C. This final temperature is maintained during 5 min. The carrier gas was premium quality N_2_ with a flux of 1 mL min^−1^ approximately.

### Theoretical details

The geometries and energies of all complexes included in this study were computed at the BP86-D3/def2-TZVPP level of theory. The complexes were fully optimized and they are true minima. The BP86 functional was employed with the latest available correction for dispersion (D3),^35^ since it is needed to describe adequately the π-stacking interactions. This level of theory is a good compromise between the size of the systems and the accuracy of the results.^36^ The calculations have been carried out by utilizing the program TURBOMOLE version 7.0.^37^ The plots of the MEP surfaces were generated using the van der Waals surface (0.001 a.u. isosurface).

## Results and discussion

### Preliminary MEP analysis

**Fig. 1:**
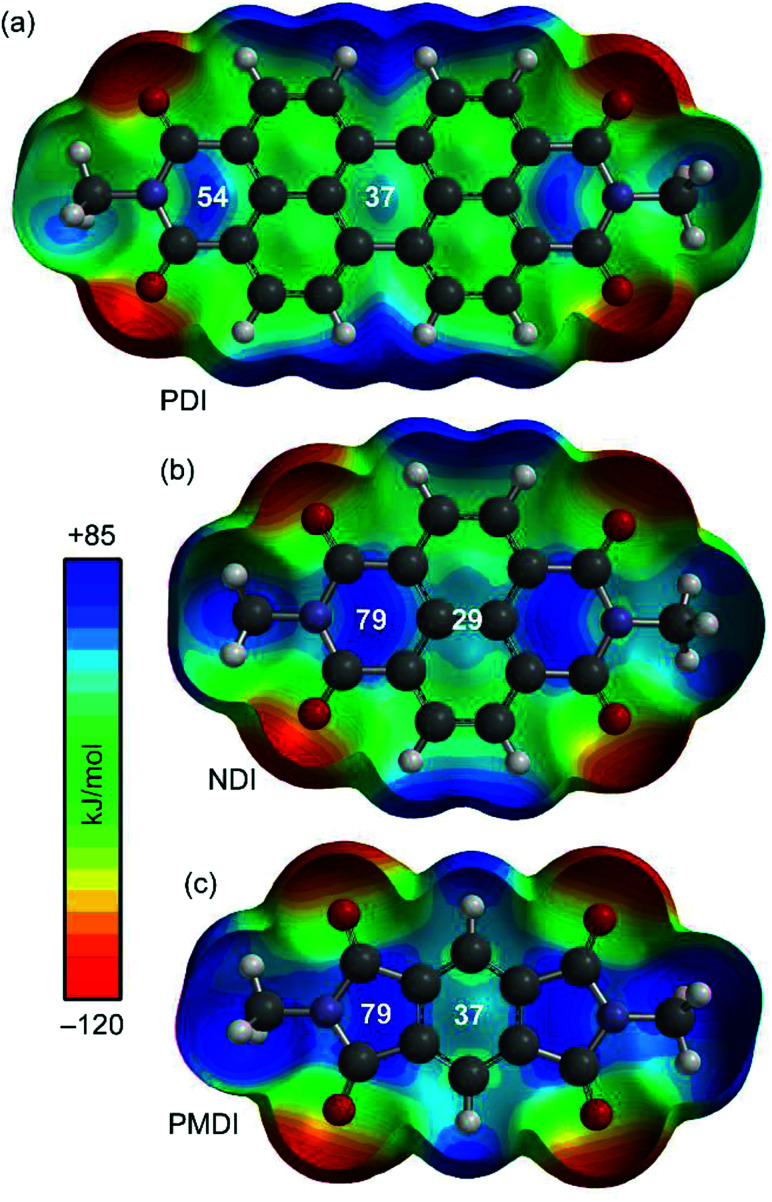
MEP surfaces of aromatic diimides 1–3 used in the study. Energies at selected points of the surface (0.001 a.u.) are given in kJ mol^−1^.

As a starting point the MEP (Molecular Electrostatic Potential) surfaces of PDI, NDI and PMDI molecules (see Fig. 1) were computed. For the three compounds, the MEP values in some selected points of the surface are indicated. It can be observed that the MEP values are more positive at the imidic rings varying from +79 for NDI and PMDI to +54 kJ mol^−1^ for PDI. The MEP values over the central rings are also positive, +37 kJ mol^−1^ for PDI and PMDI and +29 kJ mol^−1^ for NDI. Regarding the aromatic VOCs (not shown in the Fig. 1), all of them present large and negative MEP values over the center of the ring. In case of benzene the MEP value is −86 kJ mol^−1^ and the rest of aromatic ring exhibit the same value, which is slightly more negative (−90 kJ mol^−1^) in agreement with the weak electron donating nature of alkyl substituents. These preliminary analysis anticipates that benzene should have lesser affinity than the rest of aromatic VOCs. Moreover, any difference between the alkyl substituted benzenes should be due to interaction with the alkyl chain since the π-staking should be equivalent. For the aliphatic VOC, it is expected that the π-acidic surface will have affinity for the negative O atoms, although it is also possible to envisage C–H⋯O interactions since the MEP surfaces of PDI, NDI and PMDI molecules exhibit positive values in the molecular plane close to the C–H bonds (see Fig. 1).

### Energetic study

**Fig. 2:**
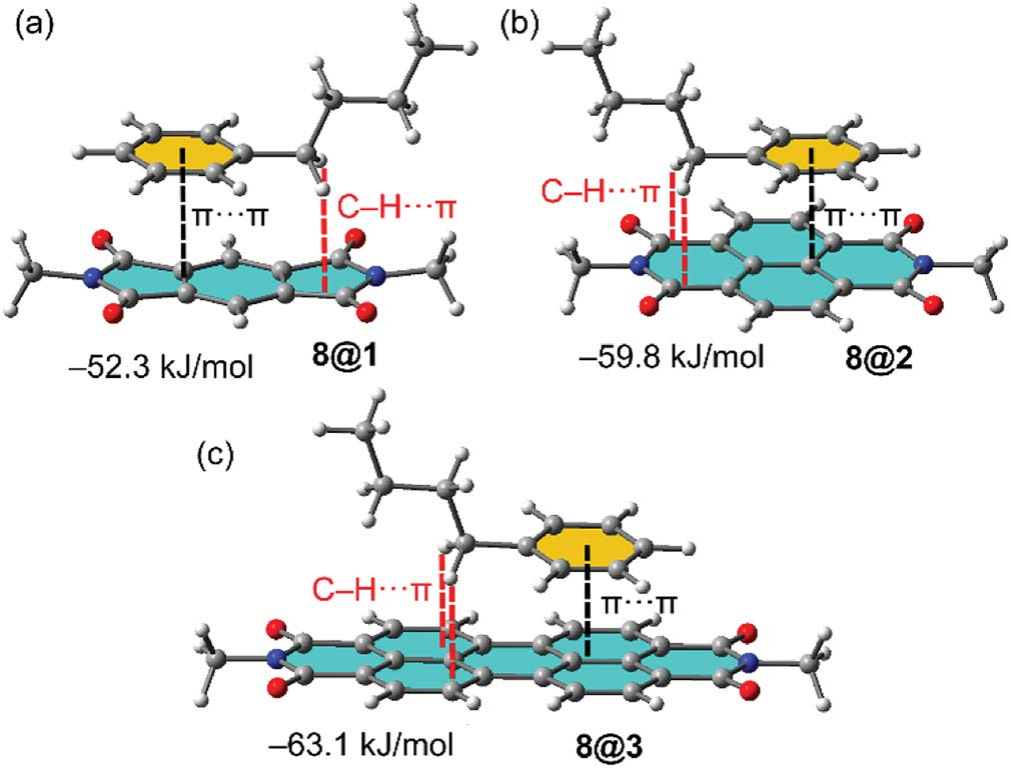
(a–c) Optimized geometries of 1 : 1 complexes between VOC 8 and the different π-acidic surfaces (PDI, NDI and PMDI) at the PB86-D3/def2-TZVPP level of theory.

In order to study the affinity of compounds 1–3 to VOCs 4–12, an energetic and geometric analysis of the complexes using DFT-D3 calculations were carried out. Different conformations and binding modes for the interaction of 1–3 with the VOCs were considered. The geometries obtained of the energetically most favourable complexes for the binding of compound 8 (as representative model of aromatic VOC) with the three receptors used in this work are given in Fig. 2. The complexes of the other aromatic VOCs are similar to those found for 8. In all cases (Fig. 2) the DFT calculations indicate that the π–π stacking complexes are the most favourable binding modes, with equilibrium distances (3.22–3.33 Å) that are in the range of strong donor–acceptor π–π interactions. Moreover, two aliphatic H-atoms also point to surface of the receptor, thus further contributing to the stabilization of the assemblies. In receptor 1 the C–H bonds are located over a five membered ring, in compound 2 over a six-membered ring and in 3 over two fused six-membered ring. Therefore, they are expected to be stronger in 3 compared to 2 and 1 because the π-system is more accessible in the former. These results suggest that the ancillary C–H⋯π interactions likely modulate the interaction energies in addition to the dominant π-stacking interaction. The binding energies are gathered in Table 1. As anticipated by the MEP analysis, among the aromatic VOCs, the benzene exhibits the lowest interaction energy for the three receptors 1–3.

**Table 1:** Interaction energies (Δ*E* in kJ mol^−1^) of complexes between receptors 1–3 and VOCs 4–12 at the PB86-D3/def2-TZVPP level of theory. The adsorption is the percentage of VOC retained in the front tube. See Scheme 1 for the structure of VOCs

VOC	Δ*E*, adsorption		
	1	2	3
4	−40.1, 59 ± 5%	−45.1, 64 ± 5%	−45.1, 65 ± 5%
5	−47.6, 62 ± 5%	−49.3, 68 ± 5%	−57.3, 76 ± 5%
6	−49.7, 69 ± 5%	−57.7, 74 ± 5%	−57.7, 75 ± 5%
7	−51.8, 70 ± 5%	−59.8, 78% ± 5%	−59.4, 77% ± 5%
8	−52.3, 70 ± 5%	−59.8, 80 ± 5%	−63.1, 89 ± 5%
9	−22.9, —a	−36.4, —a	−30.9, —a
10	−43.5, 67 ± 5%	−48.5, 69 ± 5%	−38.9, 63% ± 5%
11	−49.3, 70 ± 5%	−55.2, 75 ± 5%	−60.3, 82% ± 5%
12	−55.6, 75 ± 5%	−58.9, 81 ± 5%	−62.3, 86 ± 5%

a The presence of VOC was not detected in the front tube (see main text).

Moreover, a general trend is that the interaction energy increases as the size of the alkyl chain does. The strongest complex is established between the butylbenzene and the PDI receptor. For the aliphatic VOCs, similar trends are observed. The interaction energies increase as the size of the alkyl chain increases. Moreover, the interaction energies are in general stronger for receptor 3 apart from methyl and ethyl acetate where the PDI receptor 2 forms stronger complexes. Receptor 1 presents the weakest interaction energies in line with the smallest π-surface.

**Fig. 3:**
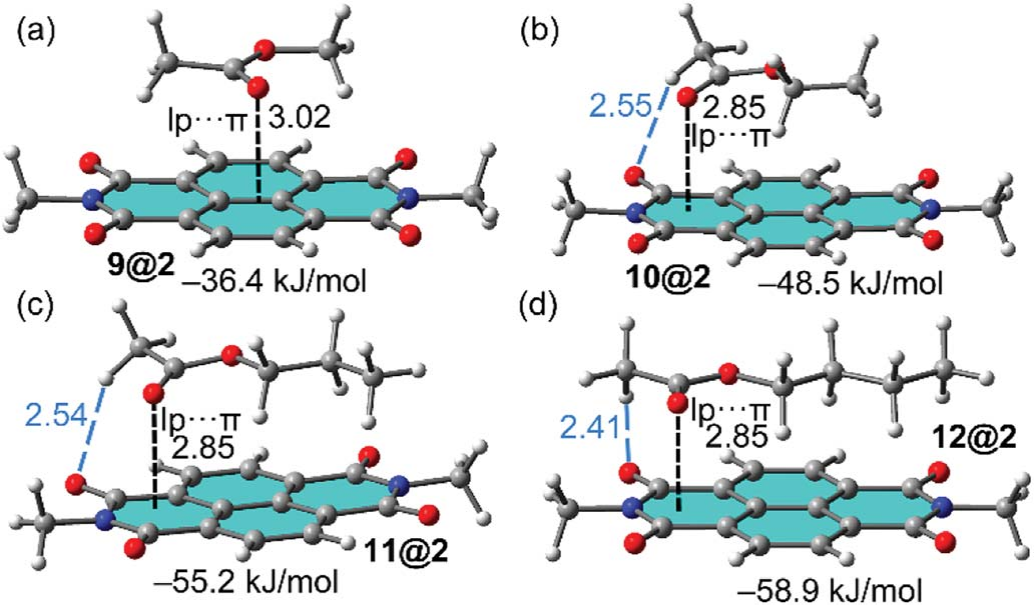
Optimized complexes of NDI 2 with methylacetate (a) and ethyl acetate (b), propylacetate (c) and butylacetate (d) at the PB86-D3/def2-TZVPP level of theory. The lone pair (lp)⋯π interactions are indicated as dashed lines. The interaction energies are also indicated.

The complexes of NDI receptor 2 with the non-aromatic VOCs are represented in Fig. 3. Apart from complex 9@2, the lp–π interaction is established with the most π-acidic imidic ring. The methyl group of acetate is located outside the π-surface of PDI moiety is such a way that one C–H bond points to the O-atom of the imide group (see blue dashed lines in Fig. 3). On the contrary, the alkyl chain is located over the π-system establishing an increasing number of C–H⋯π interactions on going from methyl to butyl, thus explaining the larger binding energies observed for VOCs 11 and 12. In addition, it can be observed that the lone pair (lp)–π interaction distance between the oxygen atom of the carbonyl group and the 6-membered imidic ring of the NDI is identical in the complexes of VOCs 10, 11 and 12 (measured from the O atom to the mean plane). Consequently, this geometric features confirms that the differences in the binding energies are due to the C–H⋯O and C–H⋯π interactions. Apart from the smallest VOC 9, the interaction energies gathered in Table 1 are similar for both aromatic and non-aromatic VOCs, therefore adsorbent material based on hybrid PMDI/NDI/PDI–Fe_3_O_4_NPs should be equally efficient for the aromatic and non-aromatic VOCs of these particular families.

**Fig. 4:**
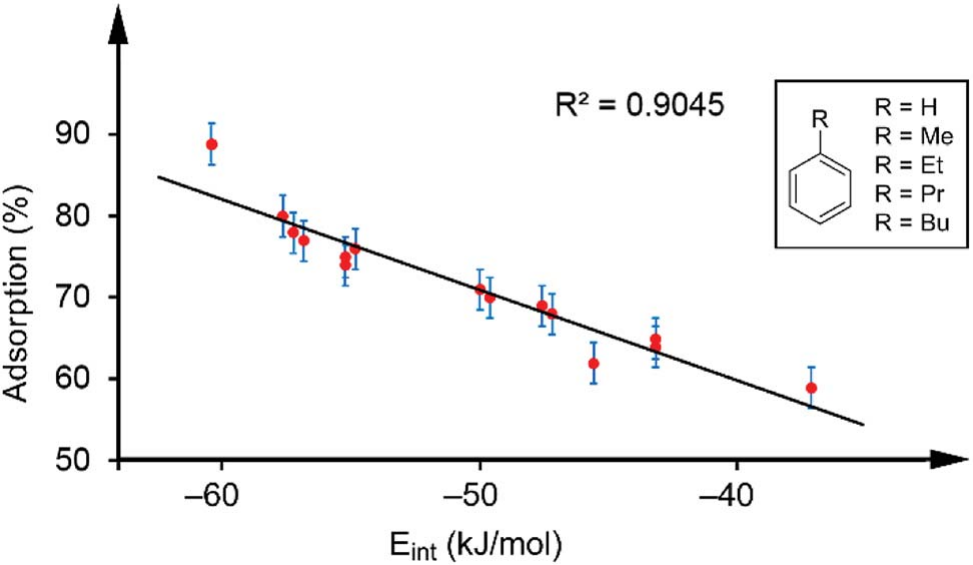
Regression plot of % quantity of VOC adsorbed and the interaction energies for the aromatic VOCs interacting with receptors 1–3.

Table 1 gathers the quantity of VOC adsorbed by the sorbent tubes based on hybrid PMDI/NDI/PDI–Fe_3_O_4_NPs. The details of how these values were obtained are detailed below. At this point, it is worthy to emphasize that for the aromatic VOCs the percentage of adsorption *versus* the interaction energies was represented. Remarkably, a good correlation between the adsorption and the interaction energies (*R*^2^ = 0.9045 see Fig. 4) was found. Such correlation strongly supports the proposed binding mode of the VOCs with the π-surface of the receptors and represents a direct correlation between the interaction energies of the π-stacked complexes and the experimental adsorption data.

### Synthesis of the magnetite supported organic–inorganic hybrids, fabrication of the sorbent tubes and adsorption–desorption experiments

For the synthesis of the magnetite supported organic–inorganic hybrids the protocol available in the literature was reproduced.^29–33^ Moreover, the synthetic protocols for the starting products perylenediimide-dopamine (PBI-DA) and pyromellitic diimide-dopamide (PMDI-DA) ligands are also available in the literature. The new naphthalendiimide-dopamine (NDI-DA) ligand was synthesized in good yield (80%) by adding two equivalents of dopamine hydrochloride to one equivalent of 1,4,5,8-naphthalentetracarboxylic dianhydride in *N*,*N*-dimethylformamide (DMF). Subsequently, the conjugation of the NDI-DA ligand with the magnetite was performed using the microwave-assisted heating method^37^ since our group has previously demonstrated for PDI-DA and PMDI-DA ligands that it increases significantly the covalent functionalization of the nanoparticle.^38^

**Scheme 2:**
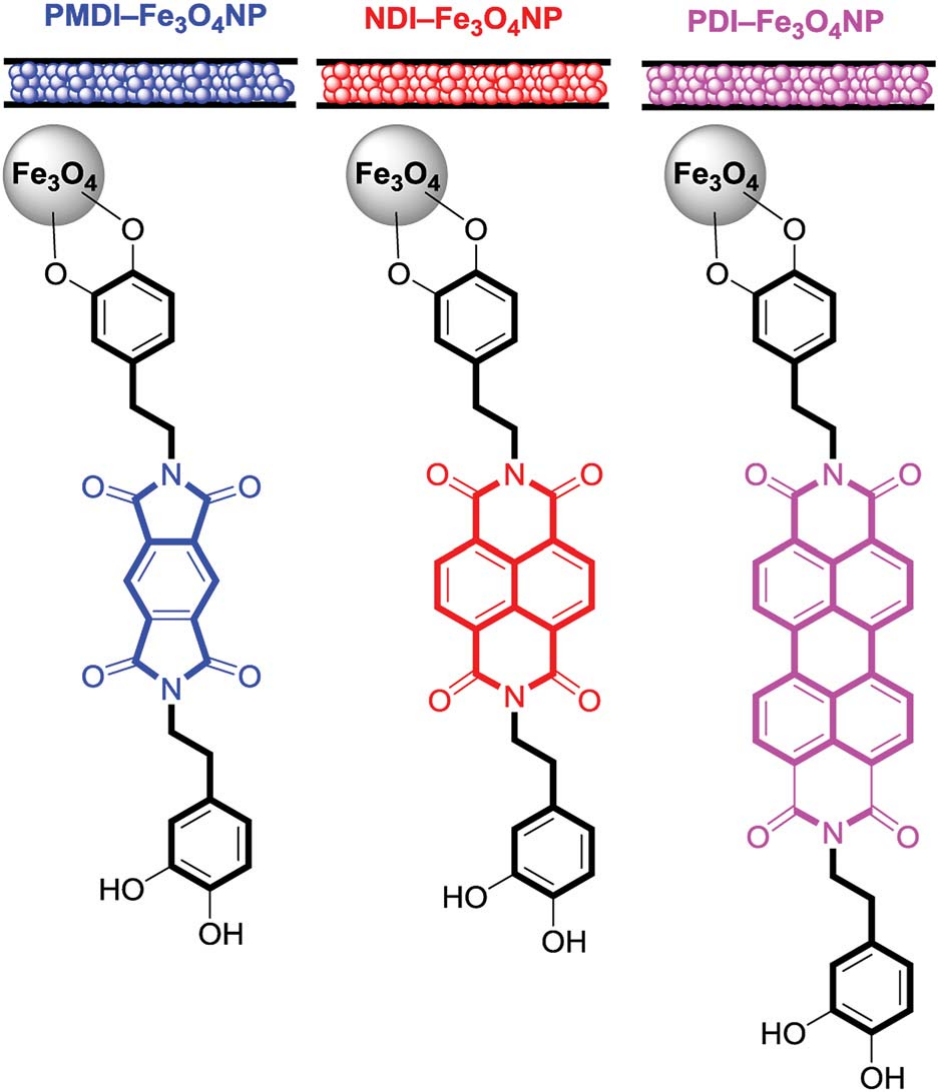
Schematic representation of the sorbent tubes and magnetite supported organic–inorganic hybrids studied in this work.

**Fig. 5:**
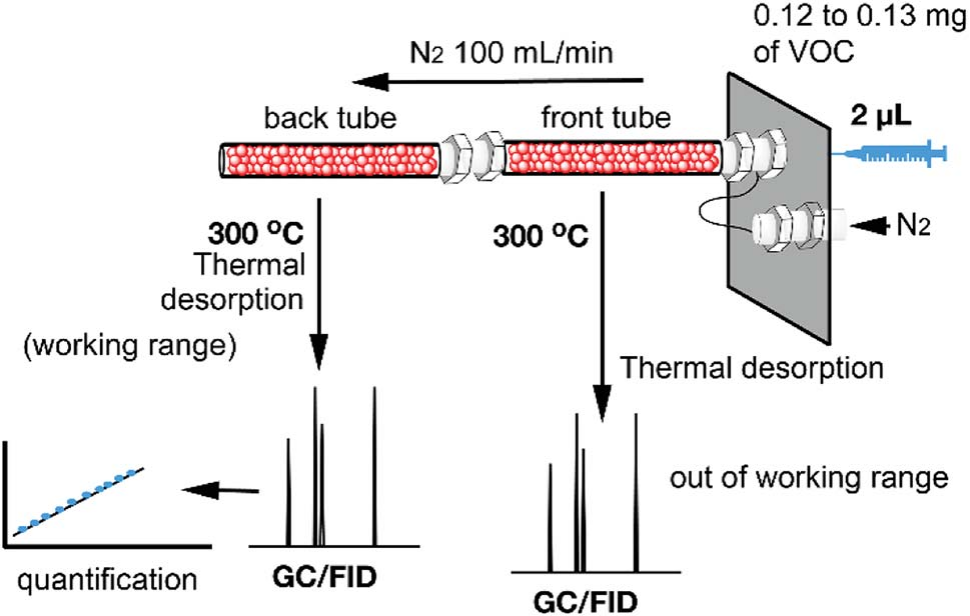
Schematic representation of the tubes connected in series. The quantitative analysis of back tube allows to quantify the amount of VOC adsorbed in the front tube.

A series of three different sorbent tubes were prepared (glass tubes of 6 mm of diameter and 90 mm of length) filled with 450 mg of the three different hybrid nanoparticles used in this work. In addition, unsilanized glass wool was placed at both ends of the tubes. Subsequently, the adsorption ability of the sorbent tubes filled with the three different hybrid Fe_3_O_4_NPs (see Scheme 2) to collect the VOCs 4–12 was studied. The procedure has been recently described our group and involves two basic steps. The first one is the utilization of one sorbent tube which is doped with different concentrations of VOCs for the generation of the working concentration range and also the calibration plots (see ref. 20 for a comprehensive description). The calibration plots and working ranges are given in the ESI.† The second step is the calculation of the adsorption capacity of the tubes. That is, two sorbent tubes connected in series (using a Swagelock-PFA union) are used, as represented in Fig. 5. Each tube is filled with 450 mg of hybrid nanoparticles, and for each experiment, a known quantity of VOC (from 0.120 to 0.130 mg) was injected, which is larger than the upper limit of the working range used for the generation of the calibration plots. The individual analysis of the back tube allows to measure the quantity of VOC that has not been adsorbed in the front tube. The results given as % VOC in the front tube are gathered in Table 1 and the absolute values are given in Table 2.

**Table 2:** Retention times (min), milligrams of injected VOC, milligrams of VOC in the back tube

VOC	RT (min)	Injected VOC (mg)	VOC in back tube^a^ (mg)
4@1	13.7	1.226	0.502 ± 0.025
4@2	13.7	1.226	0.441 ± 0.023
4@3	13.7	1.226	0.429 ± 0.020
5@1	17.5	1.214	0.461 ± 0.023
5@2	17.5	1.214	0.387 ± 0.020
5@3	17.5	1.214	0.290 ± 0.014
6@1	20.7	1.212	0.379 ± 0.019
6@2	20.7	1.212	0.315 ± 0.016
6@3	20.7	1.212	0.303 ± 0.015
7@1	23.7	1.207	0.362 ± 0.018
7@2	23.7	1.207	0.265 ± 0.013
7@3	23.7	1.207	0.277 ± 0.013
8@1	26.8	0.120	0.349 ± 0.017
8@2	26.8	0.120	0.241 ± 0.012
8@3	26.8	0.120	0.134 ± 0.007
9@1	9.7	0.130	—b
9@2	9.7	0.130	—b
9@3	9.7	0.130	—b
10@1	12.2	0.126	0.416 ± 0.021
10@2	12.2	0.126	0.391 ± 0.019
10@3	12.2	0.126	0.467 ± 0.023
11@1	15.5	0.124	0.369 ± 0.018
11@2	15.5	0.124	0.311 ± 0.015
11@3	15.5	0.124	0.219 ± 0.011
12@1	18.7	0.123	0.309 ± 0.015
12@2	18.7	0.123	0.235 ± 0.012
12@3	18.7	0.123	0.173 ± 0.009

a Data from three independent experiments.

b Calibration plots are not reproducible likely due to the low adsorption of this particular VOC.

It is worth mentioning the same experiments and same protocol were carried out using the sorbent tubes packed with the non-functionalized magnetic nanoparticles, that is without the PMDI/NDI/PDI unit. As expected, the adsorption capability of the magnetic nanoparticles decreased drastically, ranging from 40 to 60% of adsorption reduction. More importantly, no significant differences for the adsorption capacity of the magnetic NP for the different aromatic VOCs were observed, thus evidencing the important and differential feature of the π-acidic surfaces in the hybrid nanoparticles interacting with the VOCs. This result strongly agrees with the correlation found for the absorption of VOCs and the interaction energies. Moreover, several works devoted to molecular recognition based on PMDI/NDI/PDI receptors have also correlated the π-acidity of their aromatic surfaces with their ability to recognize a variety of electron rich guests.^39^

The comparison of the % VOC found in the front tube for the three different hybrid nanoparticles shows that the NDI can be a convenient aromatic receptor for the construction of adsorption tubes since it is more economic and easier to use compared to PDI. In the case of the simplest PMDI, the percentage of adsorption is reduced in almost the 20% for some VOCs (for instance butylbenzene) and consequently its utilization for the construction of sorbent tubes is not recommended.

## Conclusions

In conclusion, the capability of functionalized Fe_3_O_4_ magnetic nanoparticles to adsorb aromatic and aliphatic VOCs have been demonstrated in this work. Three different aromatic surfaces (PMDI, NDI and PDI) have been used to decorate the nanoparticles and analyze how the adsorption capacity is affected. DFT-D3 calculations were also performed that evidenced a strong correlation between the adsorption capacity of the magnetite supported organic–inorganic hybrids and the interaction energies of the π-stacked complexes, which strongly supports the binding mechanism.

This investigation also demonstrates that the NDI is a good candidate to construct hybrid materials to be used for the construction of sorbent tubes and utilized for the quantification of aromatic and aliphatic VOCs. Quite remarkably, these sorbent tubes can be desorbed and reused more than 200 times without losing their properties. The utilization of mixtures of the three hybrid nanoparticles is under investigation to analyze possible synergetic effects.

## Conflicts of interest

There are no conflicts to declare.

## Supplementary Material

RA-009-C9RA04490F-s001
