# Supplementary material for: Influence of the aromatic surface on the capacity of adsorption of VOCs by magnetite supported organic–inorganic hybrids
Source: RSC Adv. 2019 Aug 5;9(42):24184–91. doi: 10.1039/c9ra04490f (PMC9069820; doi:10.1039/c9ra04490f)

Electronic Supplementary Material (ESI) for RSC Advances.
This journal is © The Royal Society of Chemistry 2019

Electronic Supporting Information

Influence of the Aromatic Surface on the Capacity of Adsorption of VOCs by Magnetite Supported Organic-Inorganic Hybrids

María de las Nieves Piña,[a],* María Susana Gutiérrez,[a] Mario Panagos,[a] Paulino Duel,[a] Alberto León,[a] Jeroni Morey,[a] David Quiñonero[a] and Antonio Frontera[a],*

Structural Characterization

PMDI (1)

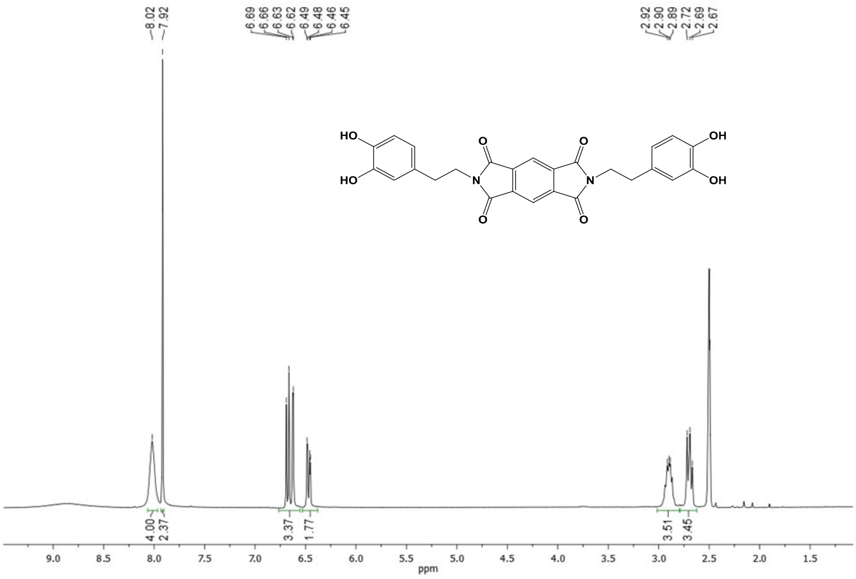

Figure S1. ^{1}H-NMR spectrum of PMDI (1) in DMSO-d_6.

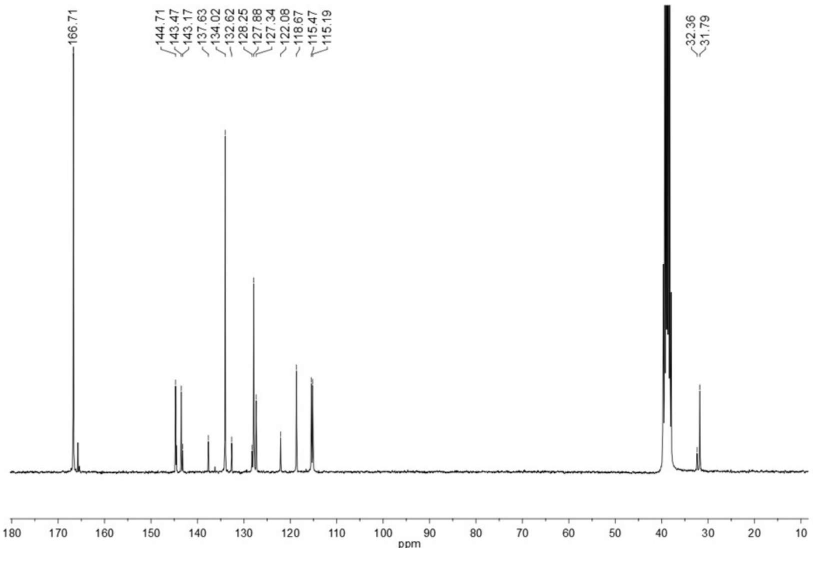

Figure S2. ^{13}C-NMR spectrum of PMDI (1) in DMSO-d_6.

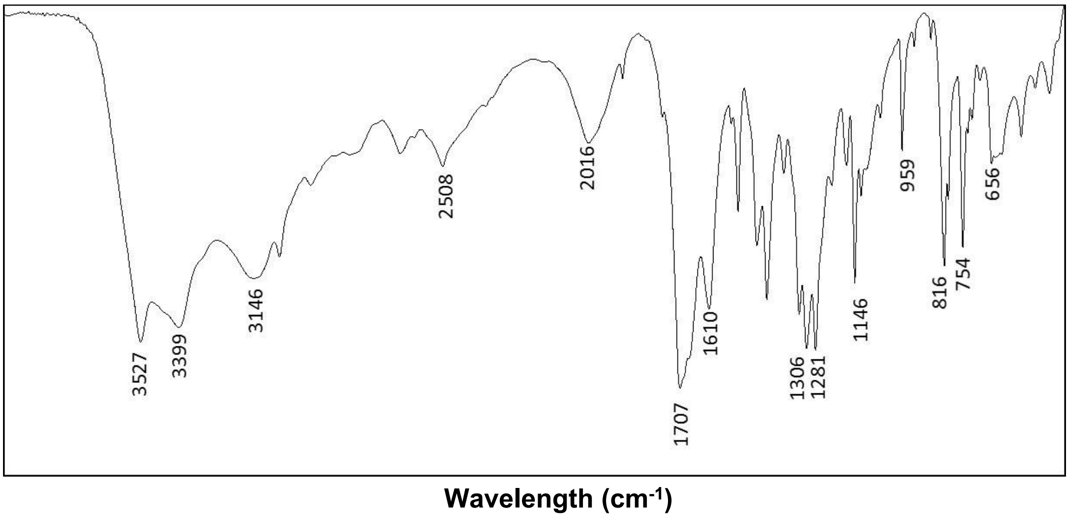

Figure S3. FTIR spectrum of PMDI (1), solid, KBr.

NDI (2)

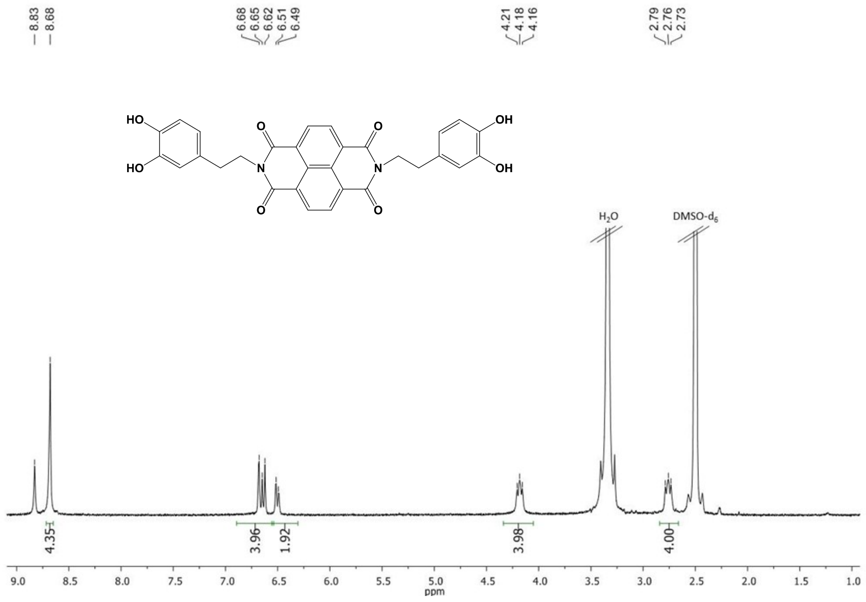

Figure S4. ^{1}H-NMR spectrum of NDI (2) in DMSO-d_6.

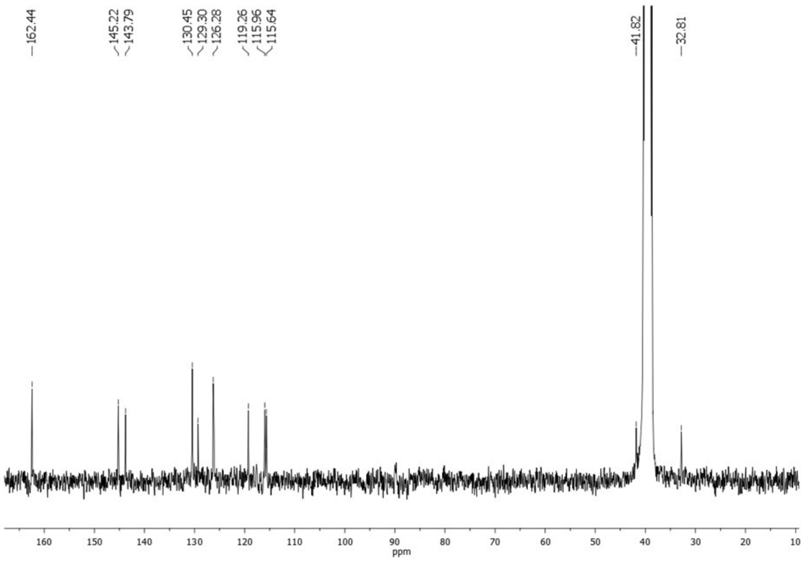

Figure S5. ^{13}C-NMR spectrum of NDI (2) in DMSO-d_6.

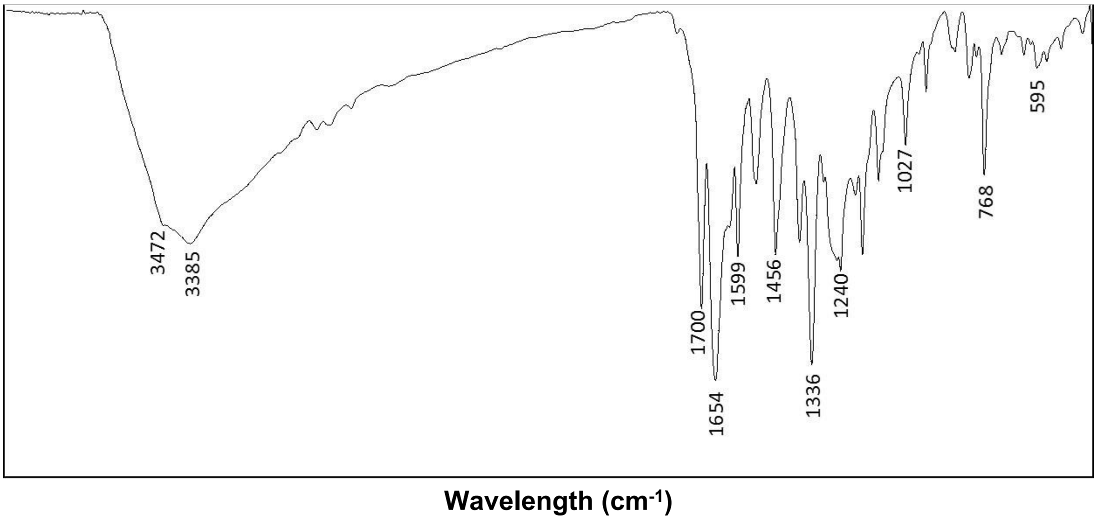

Figure S6. FTIR spectrum of NDI (2), solid, KBr.

PDI (3)

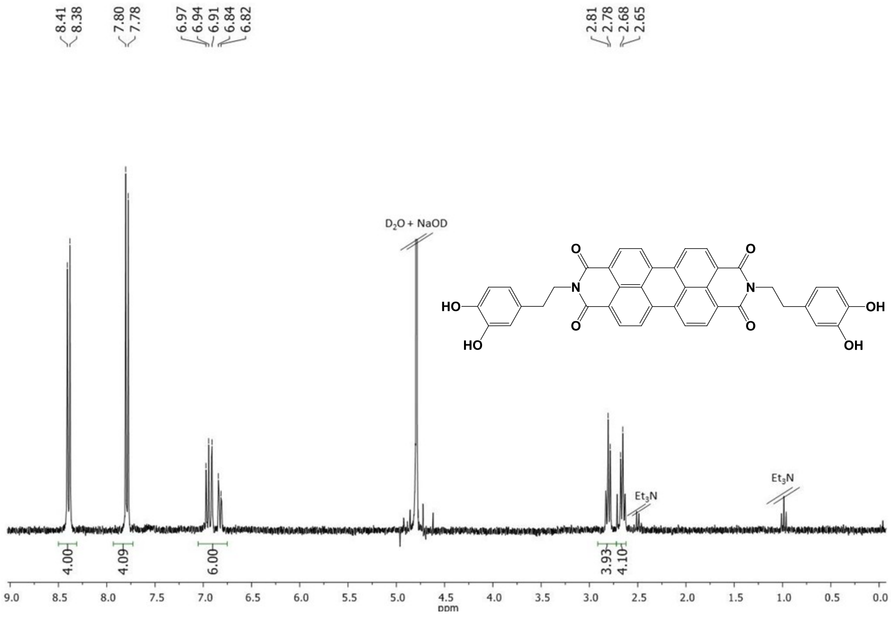

Figure S7. ^{1}H-NMR spectrum of PDI (3) in D_2O/NaOD.

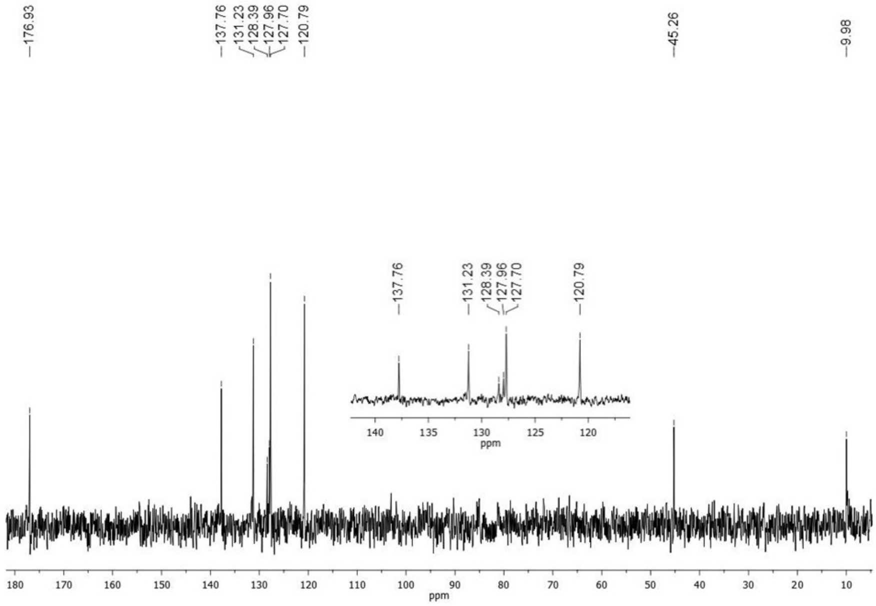

Figure S8. ^{13}C-NMR spectrum of PDI (3) in D_2O/NaOD.

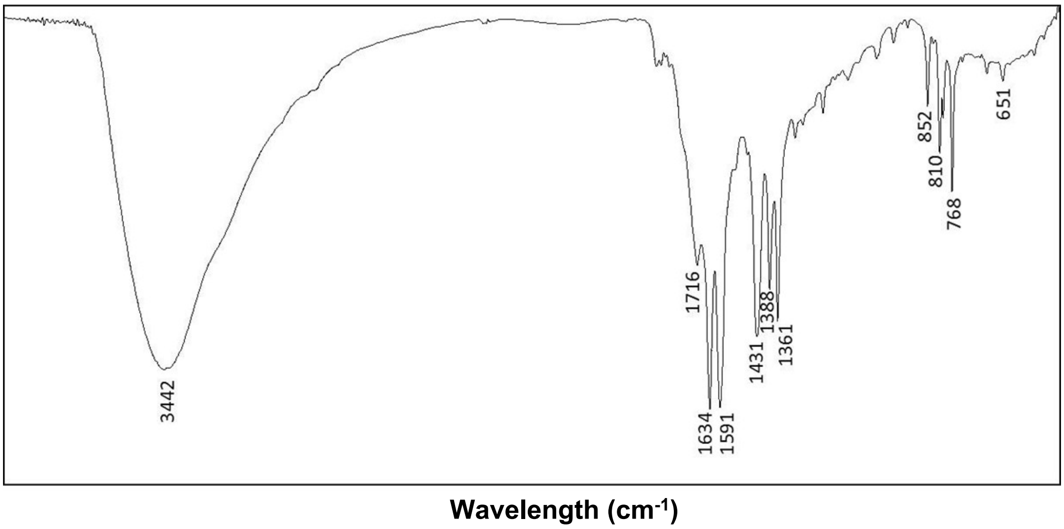

Figure S9. FTIR spectrum of PDI (3), solid, KBr.

PMDI-Fe_3O_4NP

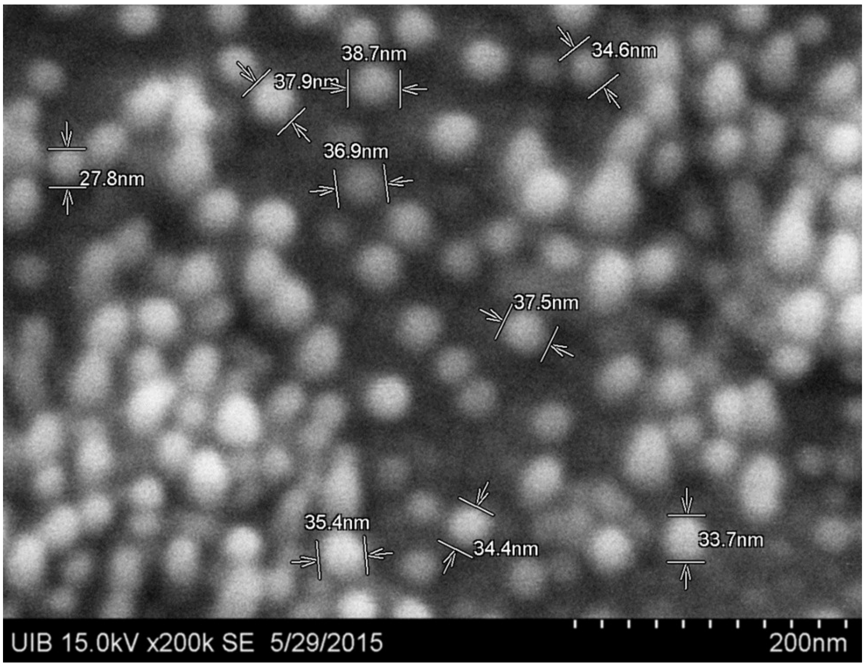

Figure S10. SEM Micrographic of PMDI-Fe_3O_4NP

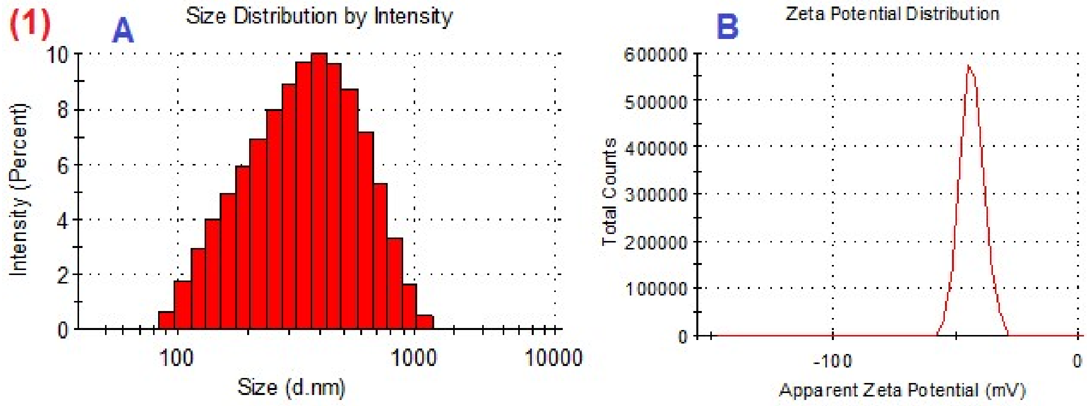

Figure S11. (A) DLS size distribution and (B) Zeta potential of PMDI-Fe_3O_4NP in H_2O (pH=7.0) at 25°C. Hydrodynamic Size = 295.0 nm. Zeta potential = -43.3 mV.

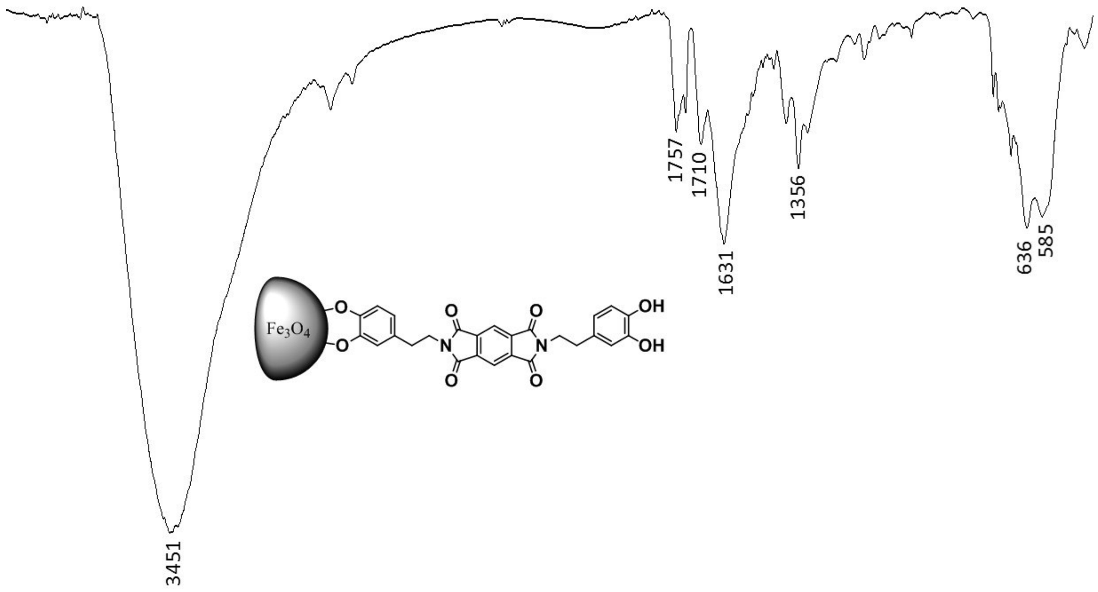

Figure S12. FTIR of PMDI-Fe_3O_4NP (KBr).

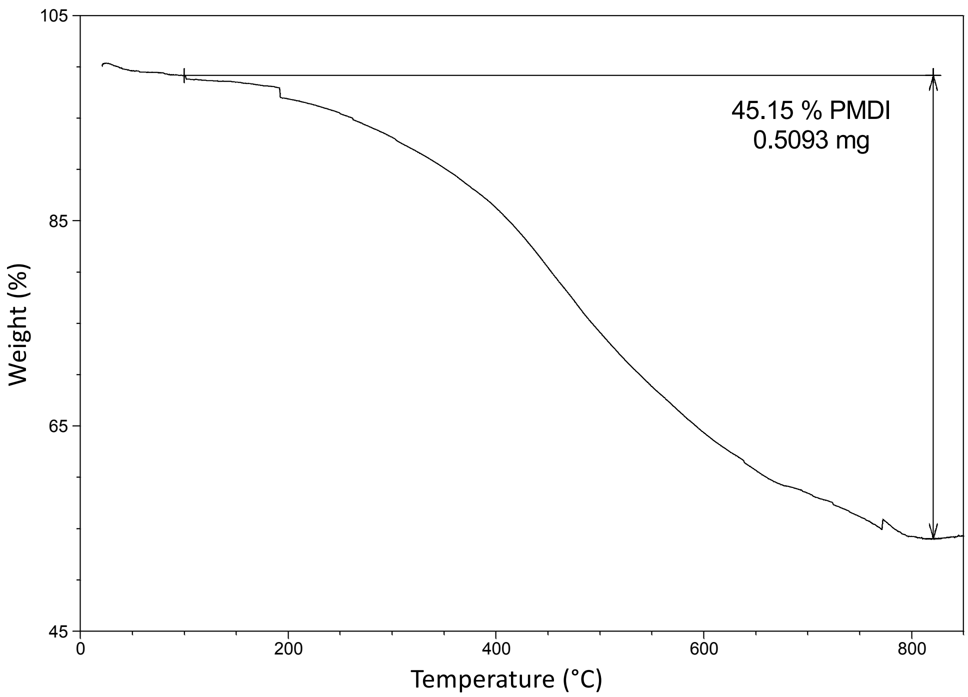

Figure S13. Thermographic analysis of PMDI-Fe_3O_4NP.

NDI-Fe_3O_4NP

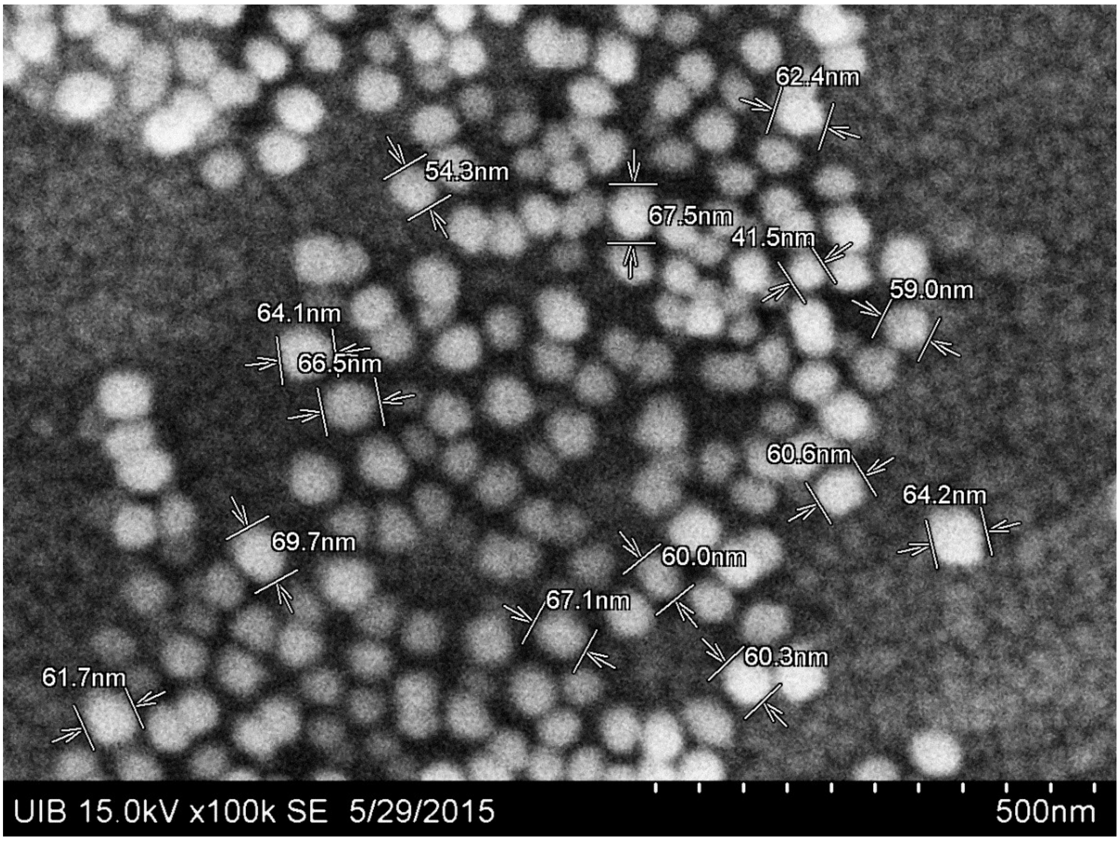

Figure S14. SEM Micrographic image of NDI-Fe_3O_4NP.

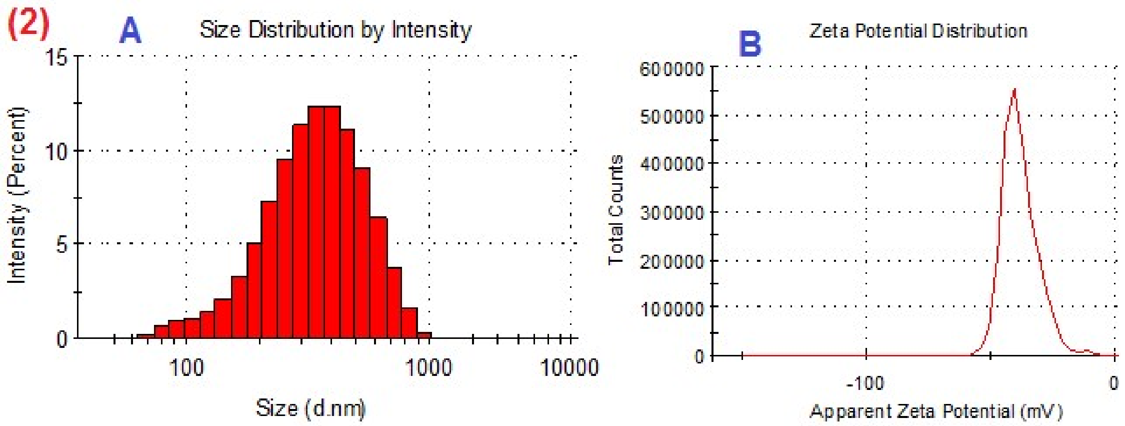

Figure S15. (A) DLS size distribution and (B) Zeta potential of NDI-Fe_3O_4NP in H_2O (pH=7.0) at 25°C. Hydrodynamic Size = 300.6 nm. Zeta potential = - 38.3 mV.

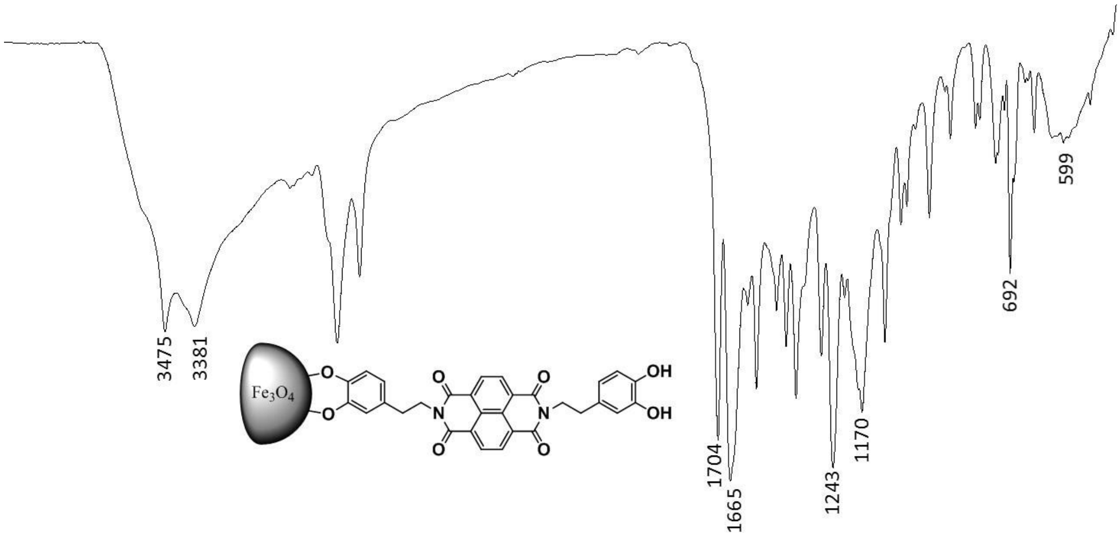

Figure S16. FTIR of NDI-Fe_3O_4NP (KBr).

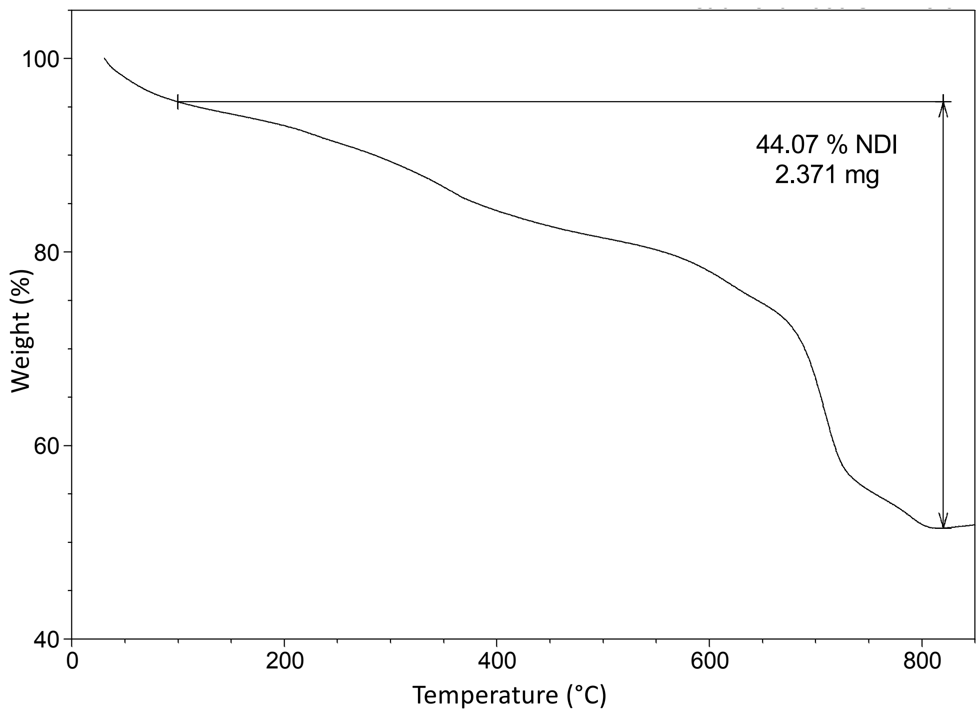

Figure S17. Thermographic analysis of NDI-Fe_3O_4NP.

PDI-Fe_3O_4NP

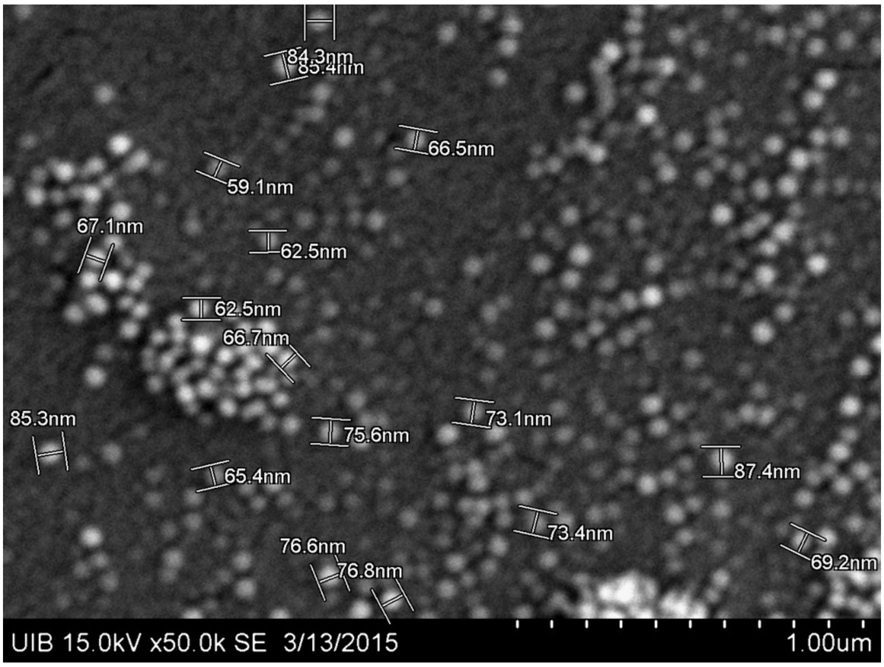

Figure S18. SEM Micrographic of PDI-Fe_3O_4NP

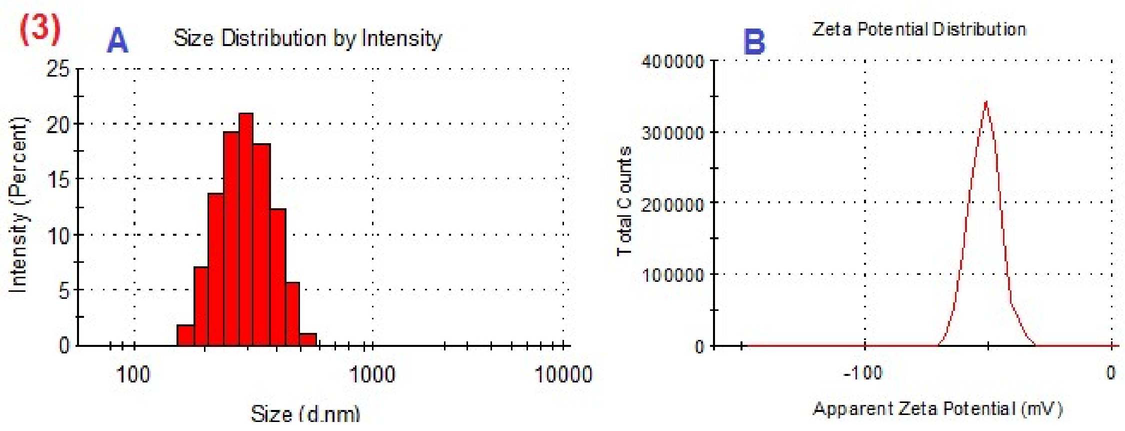

Figure S19. (A) DLS size distribution and (B) Zeta potential of NDI-Fe_3O_4NP in H_2O (pH=7.0) at 25°C. Hydrodynamic Size = 335.2 nm. Zeta potential = - 51.3 mV.

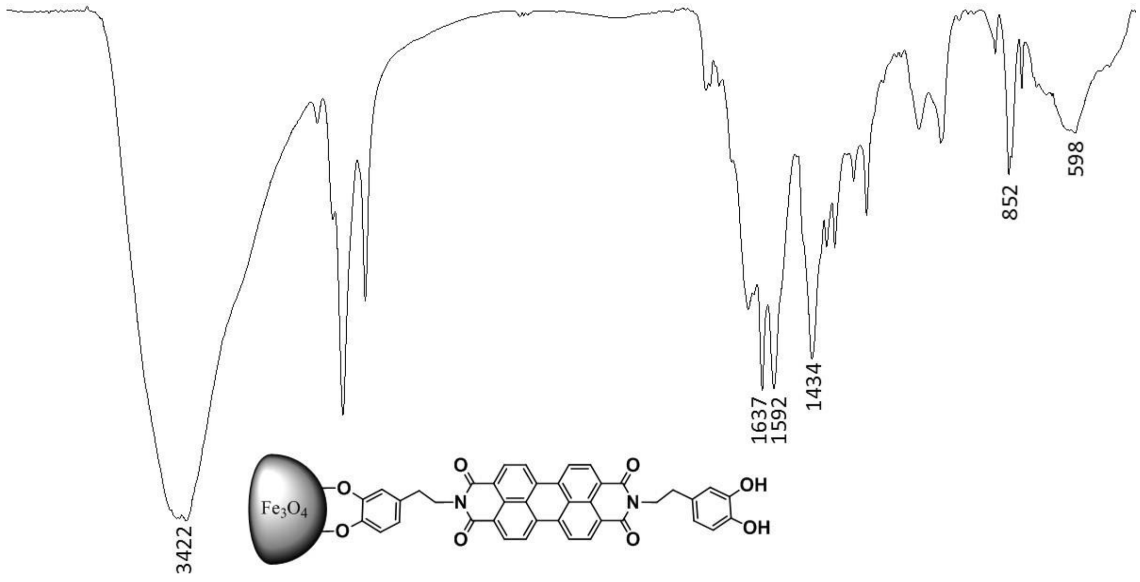

Figure S20. FTIR of PDI-Fe_3O_4NP (KBr).

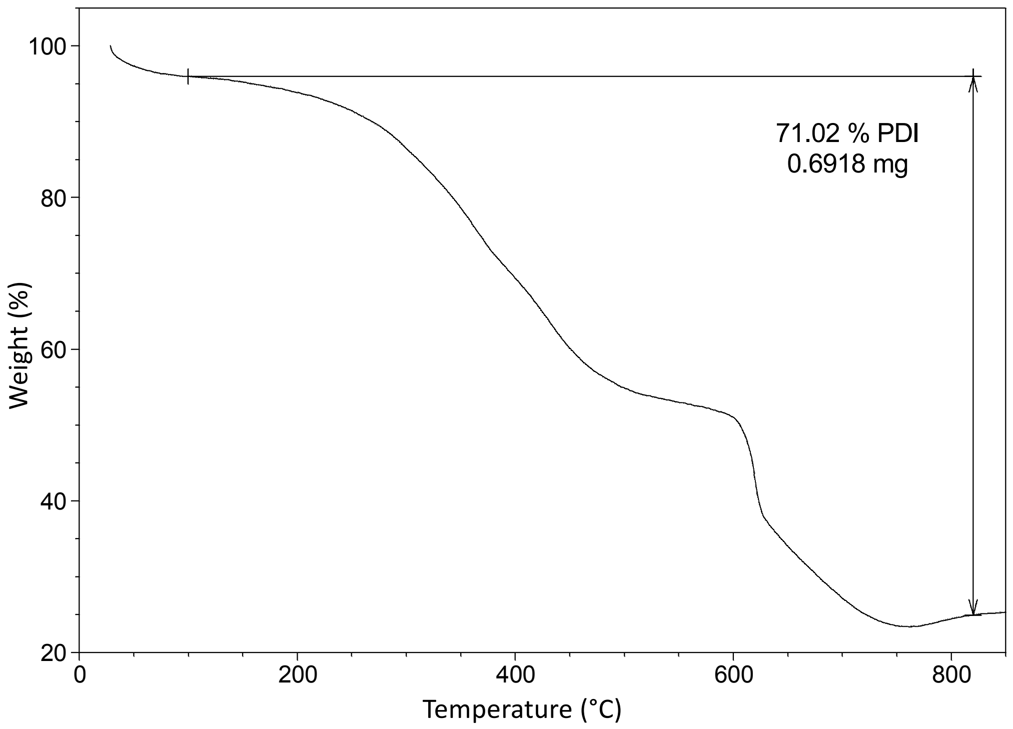

Figure S21. Thermographic analysis of PDI-Fe_3O_4NP.

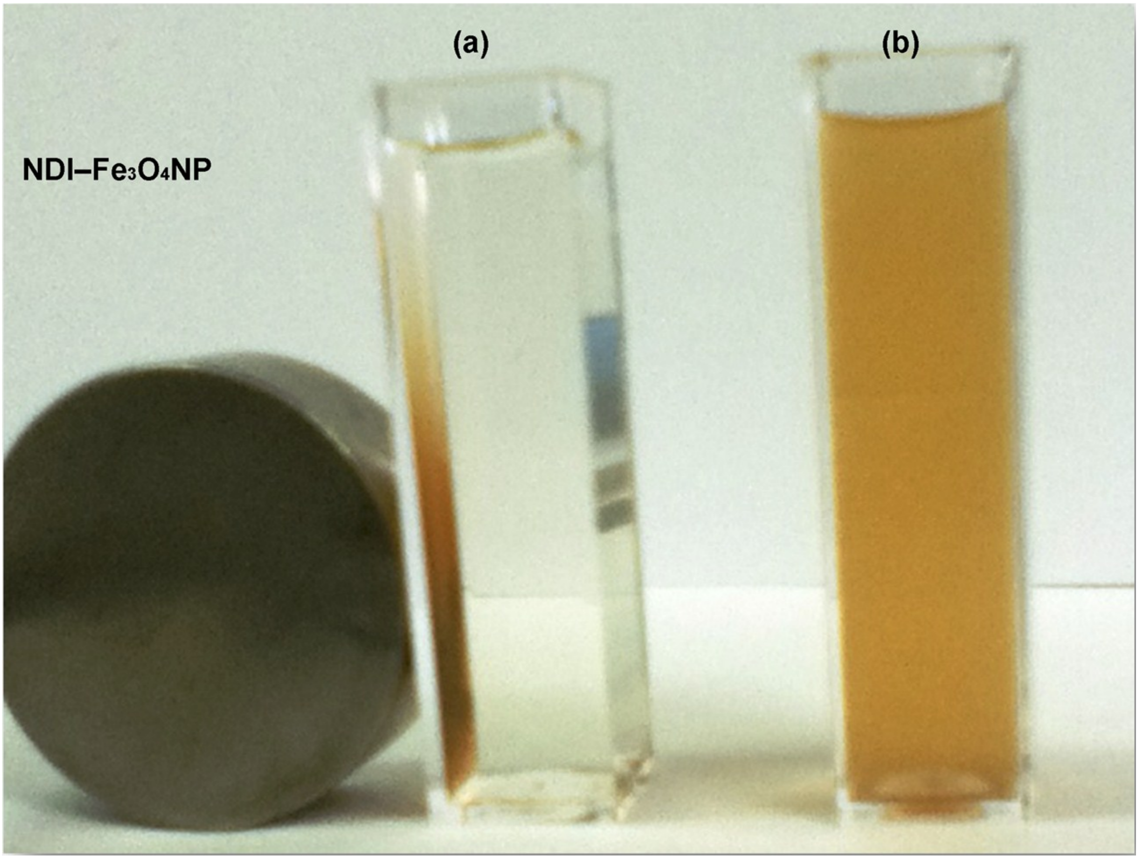

Figure S22. NDI hybrid nanoparticle in the presence (a) and absence (b) of the neodymium magnet.

Determination of number of molecules on FeNPs surface.

TGA curves shows two typical desorption zones for Fe_3O_4NP: the first zone (between 300-500 ºC) corresponds to physiosorbed molecules, the second zone (between 500-1000 ºC) corresponds to chemisorbed molecules, the summary of this two desorption zones is used to determinate the total number of molecules on the surface. Applying the following equations, the number of molecules on a Fe_3O_4NPs surface can be determinated:

$$N = \frac{\pi D^3 \rho}{6mw}$$

$$\frac{1}{N} = \frac{nanoparticles}{mol\, Fe_3O_4}$$

Where *1/N* refers to the number of Fe_3O_4NP for each mol of Fe_3O_4. *D* is the average diameter of Fe_3O_4NP in cm (provided by TEM micrographs), ρ is Fe_3O_4 density (5.196 g/cm^3) and *mw* is the molecular weight of Fe_3O_4 (231.53 g/mol). In order to improve and clarify the number of substituents, we chose TGA method to determinate conjugation rate against mass loss due to decomposition. Experiments were performed with constant heating rate of 10 ºC/min from room temperature (25 ºC) to 1000 ºC. Using weight loss percentage values it is possible to quantify the number of molecules on Fe_3O_4NP surface applying previous equations.

	Weight loss (%)	Molecules on Fe_3O_4NP surface	ξ (molecules per nm^2)
**PMDI-Fe_3O_4NP**	45.15	6.26 x 10^{17}	55.3
**NDI-Fe_3O_4NP**	47.07	2.65 x 10^{18}	74.1
**PDI-Fe_3O_4NP**	71.02	6.28 x 10^{17}	89.3

Table S1. Thermogravimetric values of Fe_3O_4-NPs.

	BET surface (m^2/g)	Maximum absorption (cm^3/g)	Pore diameter (nm)
**PMDI-Fe_3O_4NP**	78.39	181.0	15.1
**NDI-Fe_3O_4NP**	79.07	233.0	19.0
**PDI-Fe_3O_4NP**	20.67	52.0	17.5

Table S2. Values for specific surface BET essays of Fe_3O_4-NPs.

Calibration Plots

In the following figures we represent the calibration plots obtained for each analyte (VOC). We represent the range of concentration that the linear behavior

is maintained. Higher concentrations provoke the saturation of the tube and were used for the experiments where two tubes were connected in series and to measure the % of retention.

PMDI-Fe_3O_4NP

Benzene (4)

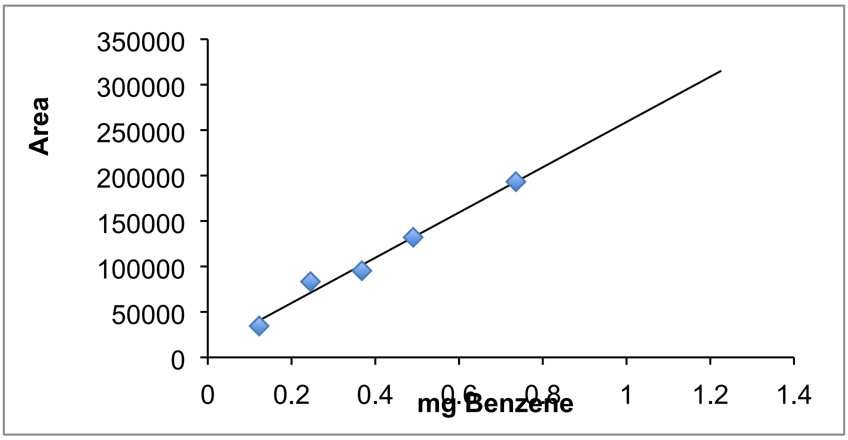

Toluene (5)

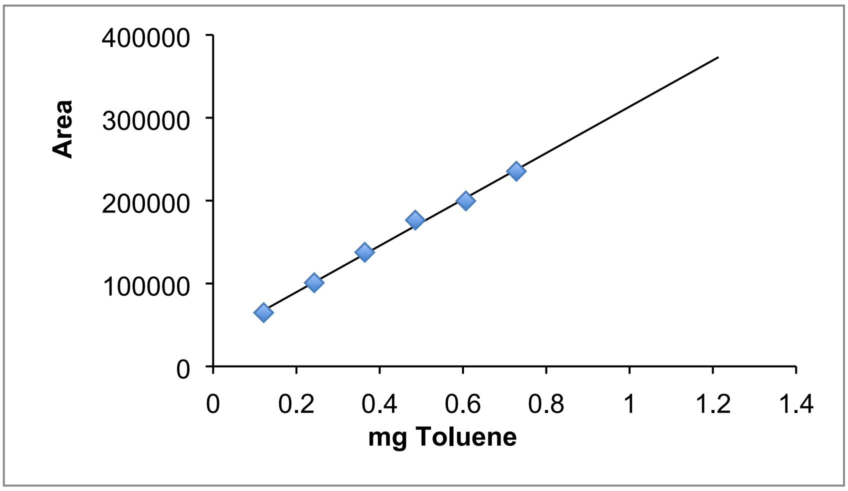

Ethyl benzene (6)

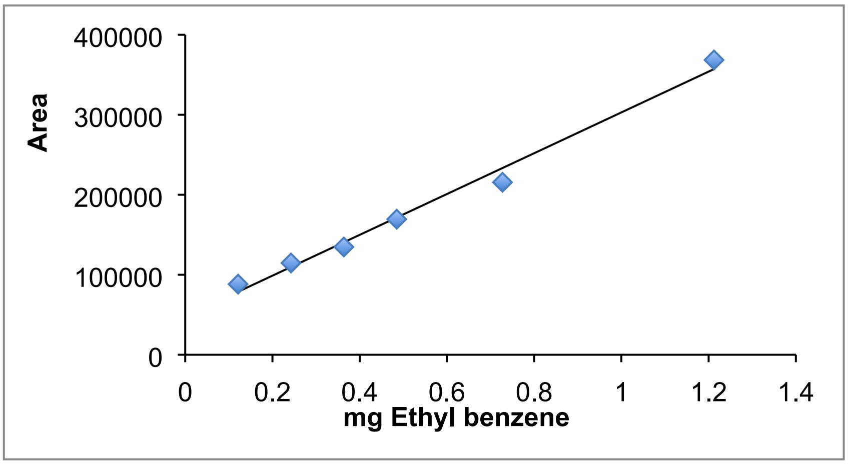

Propyl benzene (7)

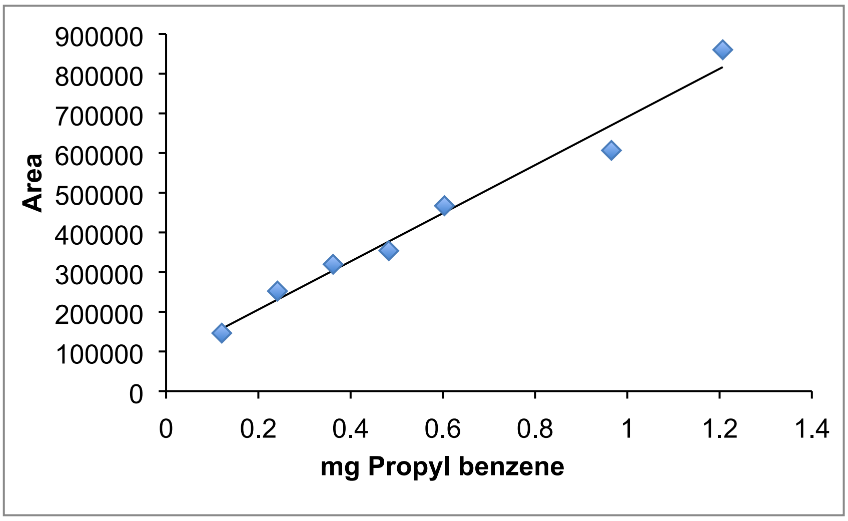

Butyl benzene (8)

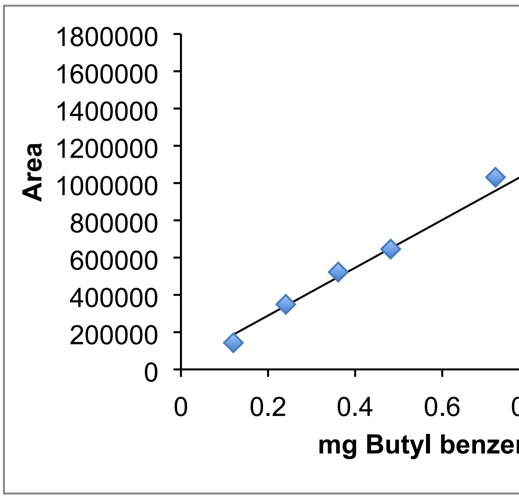

Ethyl acetate (10)

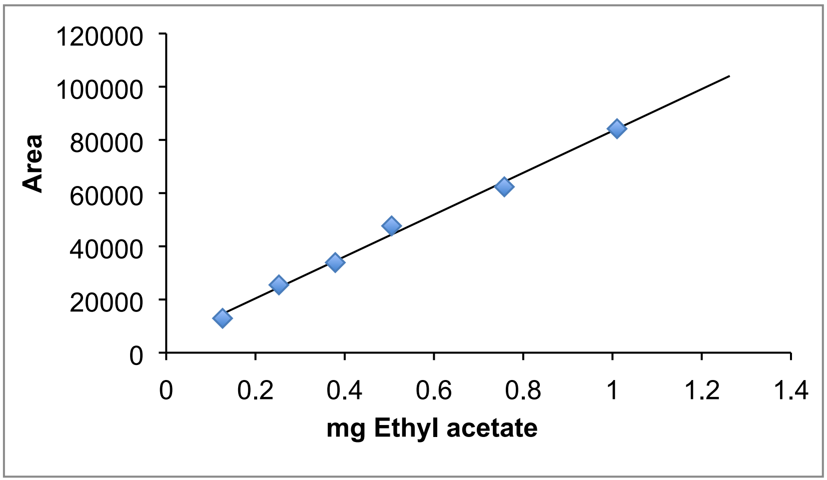

Propyl acetate (11)

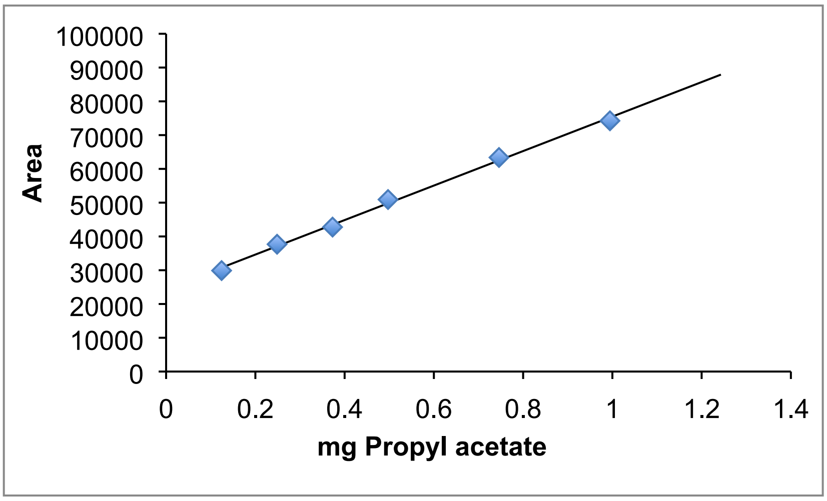

Butyl acetate (12)

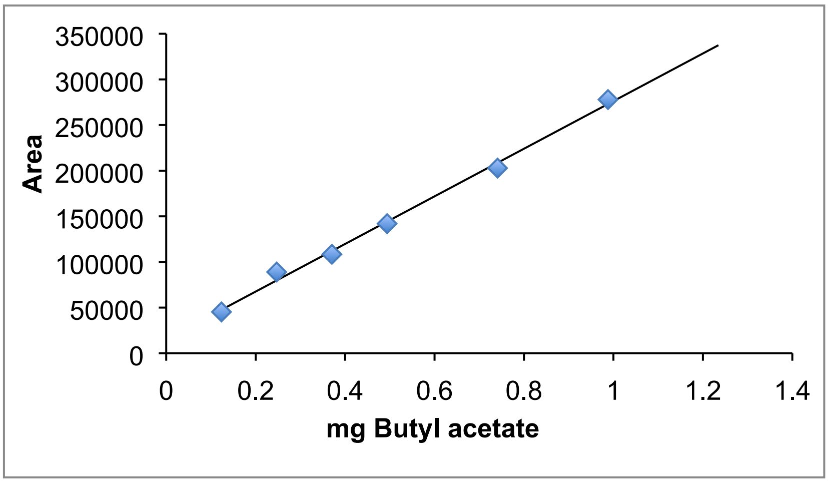

NDI-Fe_3O_4NP

Benzene (4)

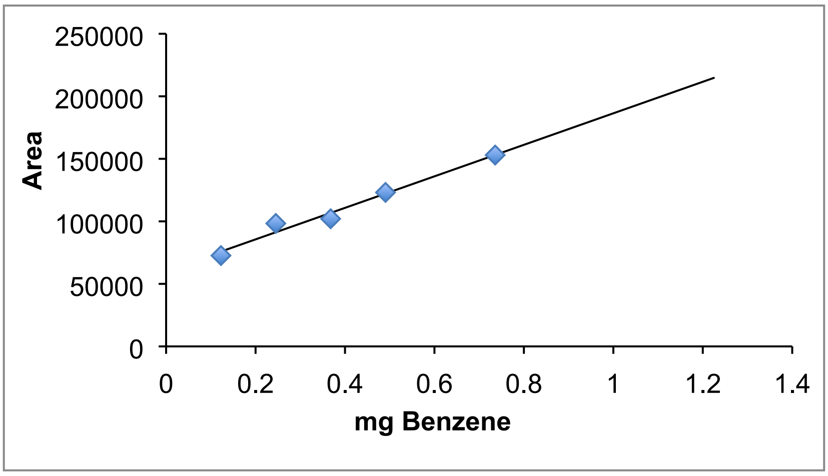

Toluene (5)

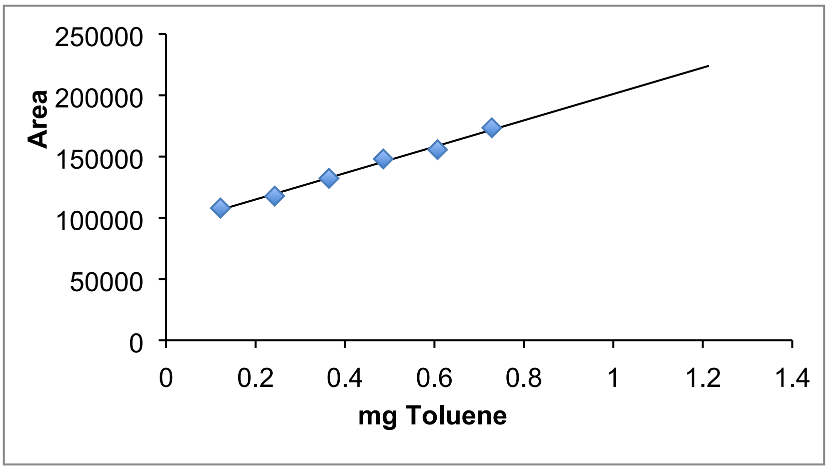

Ethyl benzene (6)

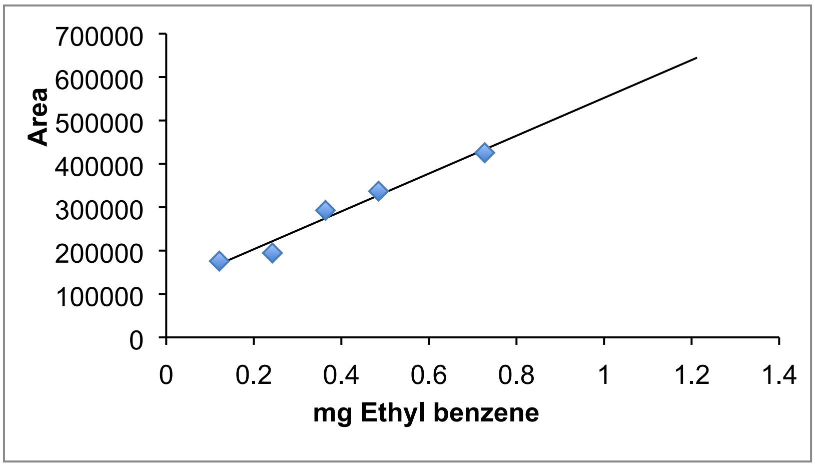

Propyl benzene (7)

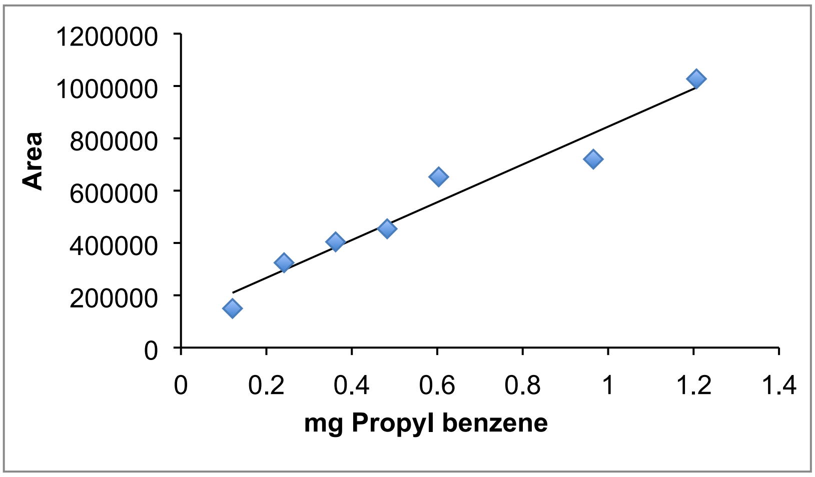

Butyl benzene (8)

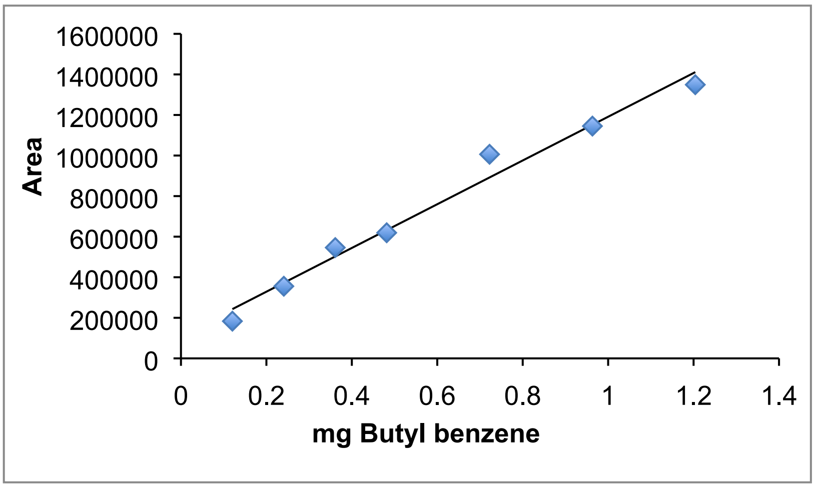

Ethyl acetate (10)

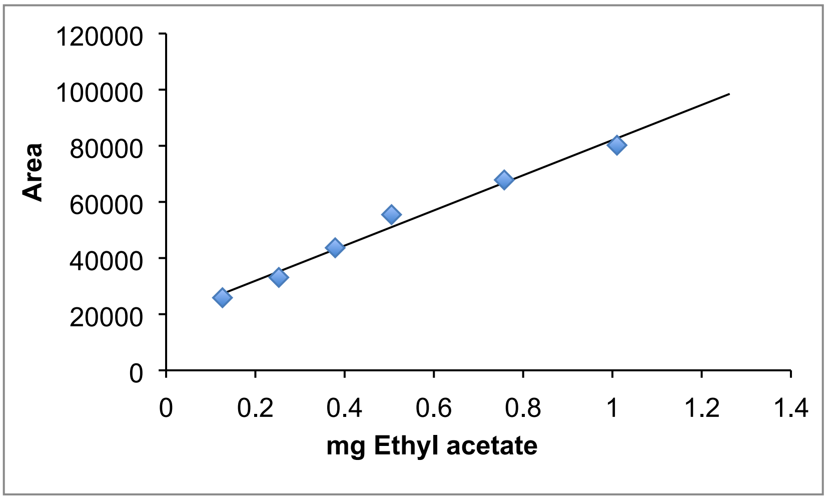

Propyl acetate (11)

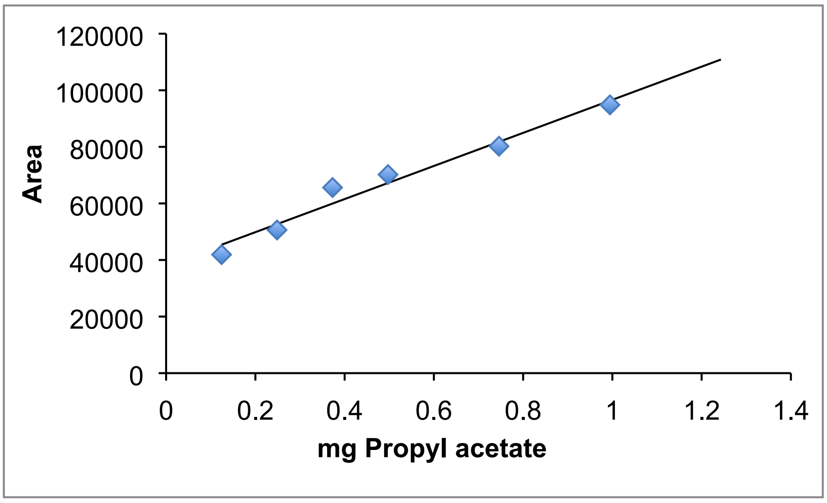

Butyl acetate (12)

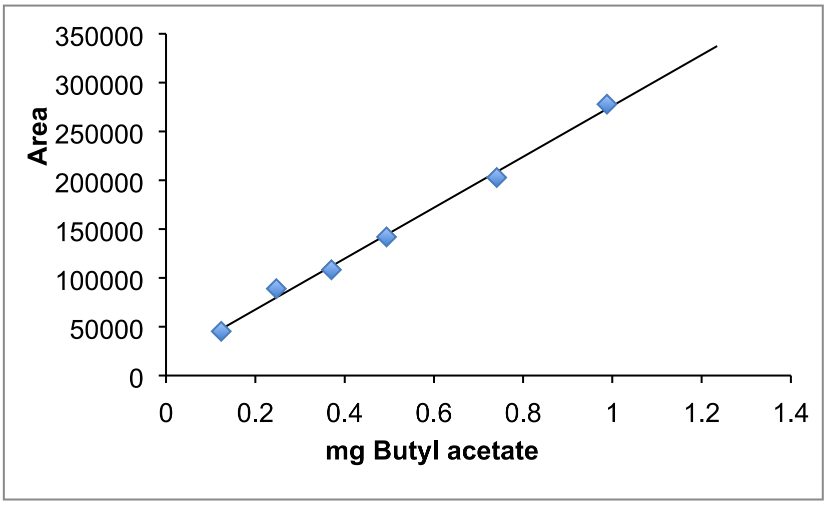

PDI-Fe_3O_4NP

Benzene (4)

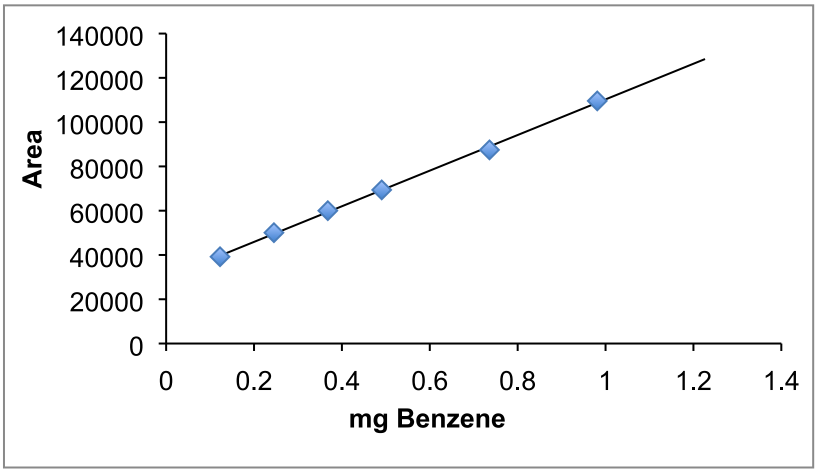

Toluene (5)

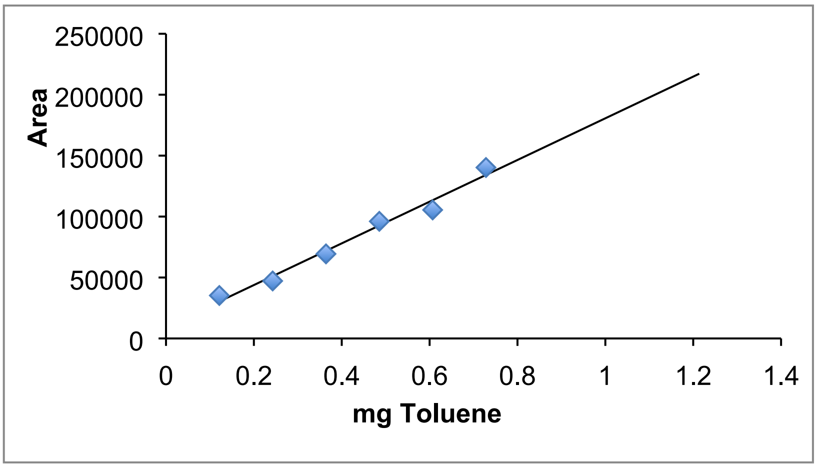

Ethyl benzene (6)

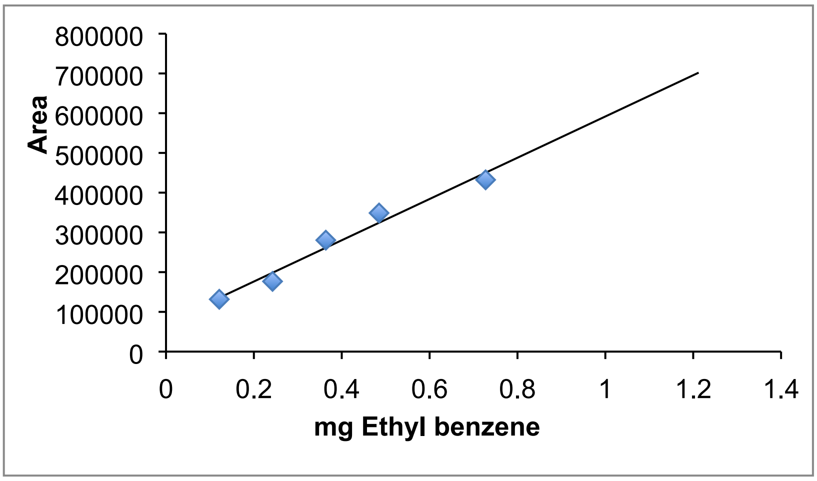

Propyl benzene (7)

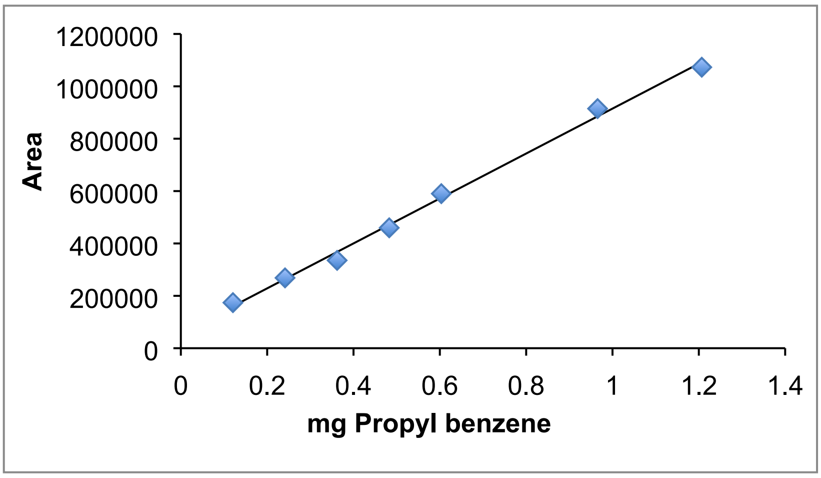

Butyl benzene (8)

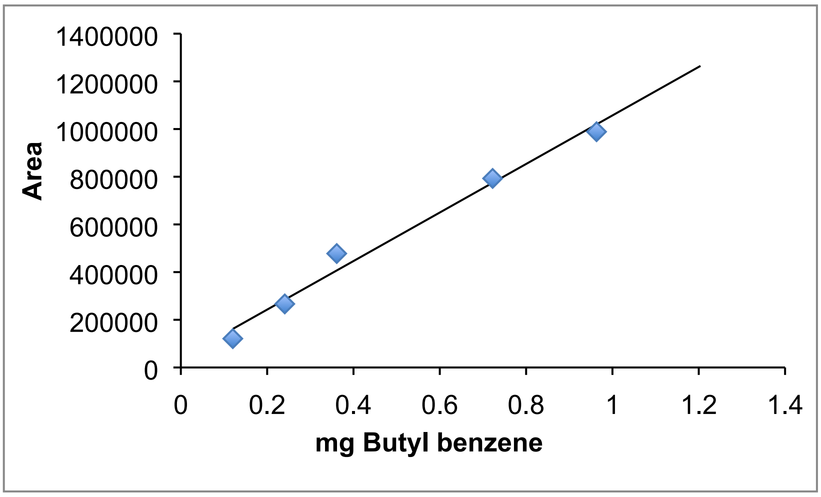

Ethyl acetate (10)

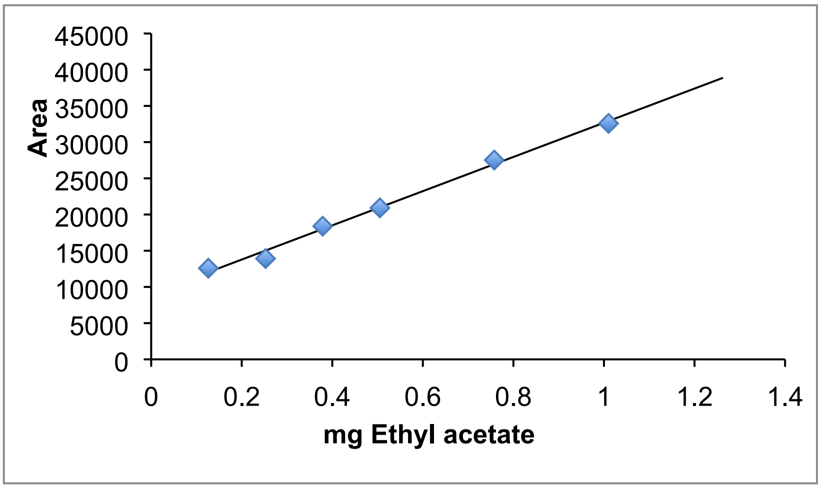

Propyl acetate (11)

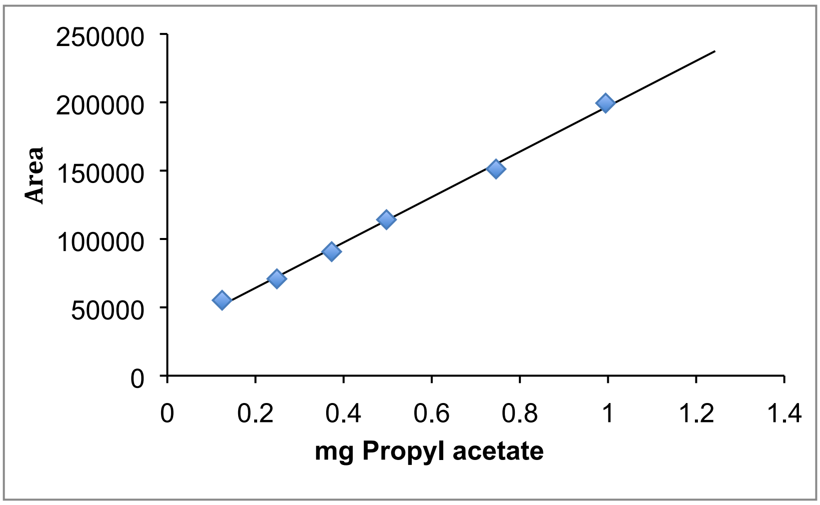

Butyl acetate (12)

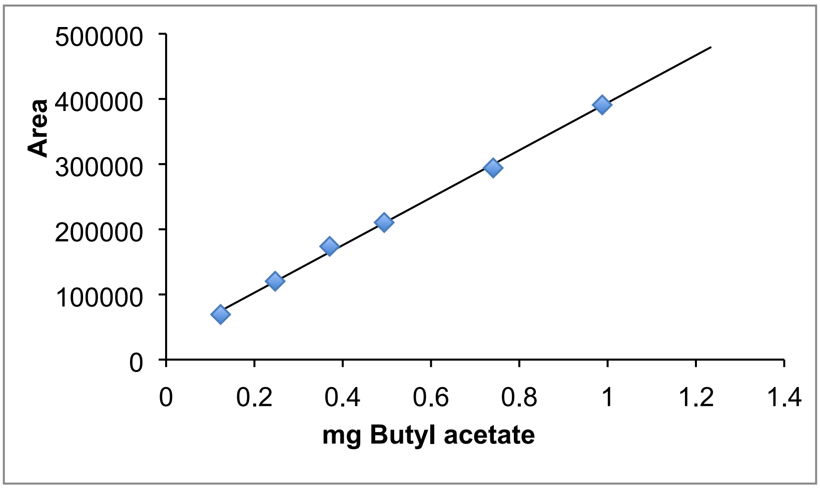

Supplement: RA-009-C9RA04490F-s001 [file RA-009-C9RA04490F-s001.pdf]
